# Efficient additive-free formic acid dehydrogenation with a NNN–ruthenium complex†

**DOI:** 10.1039/d3cy00512g

**Published:** 2023-07-17

**Authors:** Pascal Knörr, Nicolas Lentz, Martin Albrecht

**Affiliations:** a Department of Chemistry, Biochemistry & Pharmaceutical Sciences, University of Bern Freiestrasse 3 3012 Bern Switzerland martin.albrecht@unibe.ch

## Abstract

A new ruthenium complex containing a pyridylidene amine-based NNN ligand was developed as a catalyst precursor for formic acid dehydrogenation, which, as a rare example, does not require basic additives to display high activity (TOF ∼10 000 h^−1^). Conveniently, the complex is air-stable, but sensitive to light. Mechanistic investigations using UV-vis and NMR spectroscopic monitoring correlated with gas evolution profiles indicate rapid and reversible protonation of the central nitrogen of the NNN ligand as key step of catalyst activation, followed by an associative step for formic acid dehydrogenation.

## Abstract 2

A new phosphine-free *N*,*N*,*N*-coordinated ruthenium complex featuring a pyridylidene amine (PYE) ligand catalyzes the dehydrogenation of formic acid with high activity; mechanistic studies reveal a critical role of the central N-donor of the NNN ligand.

## Graphical abstract

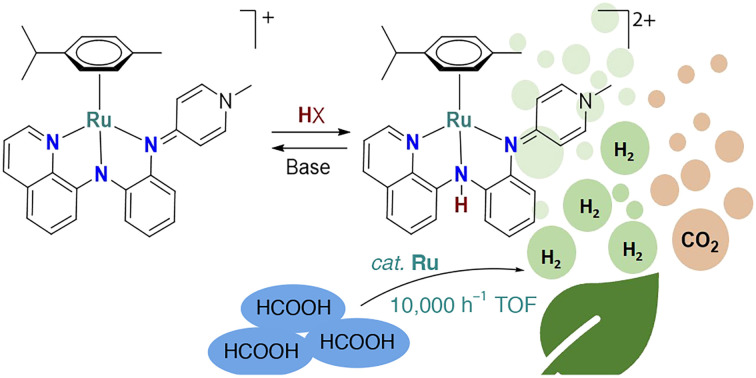

## Introduction

Storage of renewable energy is one of the key challenges to move towards a cyclic green energy economy and therefore huge efforts are being devoted towards the development of alternative fuels.^1–3^ In particular, dihydrogen has been proposed as an ideal energy carrier, as it is accessible through water splitting and only yields benign products upon oxidation in a fuel cell.^4^ To avoid the significant downsides of gaseous dihydrogen such as explosivity and low volumetric energy density, the usage of liquid organic hydrogen carriers (LOHCs) as a transient storage vector is highly promising.^5^ Small organic molecules can undergo reversible hydrogenation and dehydrogenation cycles and thus allow hydrogen to be safely stored and used.

Formic acid (HCOOH, FA) is one of the most promising liquid organic hydrogen carriers as it features high volumetric energy density, low toxicity and is liquid at room temperature.^6–8^ Hence, the dehydrogenation of FA to selectively release dihydrogen has been intensely investigated since the seminal work of Beller and Laurenczy in 2008.^9–11^ Impressive results have been achieved with homogeneous systems, even though many are compromised by insufficient productivity in the absence of basic additives.^12–25^ Notable exceptions include the systems of Li^26^ and Milstein^27^ which are active in aqueous solution and neat FA, respectively, and reach turnover frequencies (TOFs) of 487 500 and 3000 h^−1^ with turnover numbers (TONs) around 2 000 000. Solid supported heterogeneous systems have this far not reached the performance of homogeneous systems, with TOFs around 7000 h^−1^.^28^ However, these systems stand out for their extraordinary durability, which allowed the design of a continuous dehydrogenation system without detectable catalyst deactivation.^29^

The introduction of cooperative basic functionalities on the ligands has been proposed as an approach to avoid basic additives and to increase catalytic efficiency.^12,30–33^ We therefore hypothesized that efficient FA dehydrogenation catalysts may result from a ligand system that combines a basic amide site with an electronically flexible pyridylidene amine (PYE) moiety, especially since pyridylidene amines and amides have shown to promote catalytic redox processes.^13,34,35^ Herein, we report a tridentate ruthenium NNN complex featuring such a ligand design and demonstrate the role of the central amide as an internal base to ensue high catalytic activity.

## Results and discussion

**Scheme 1:**
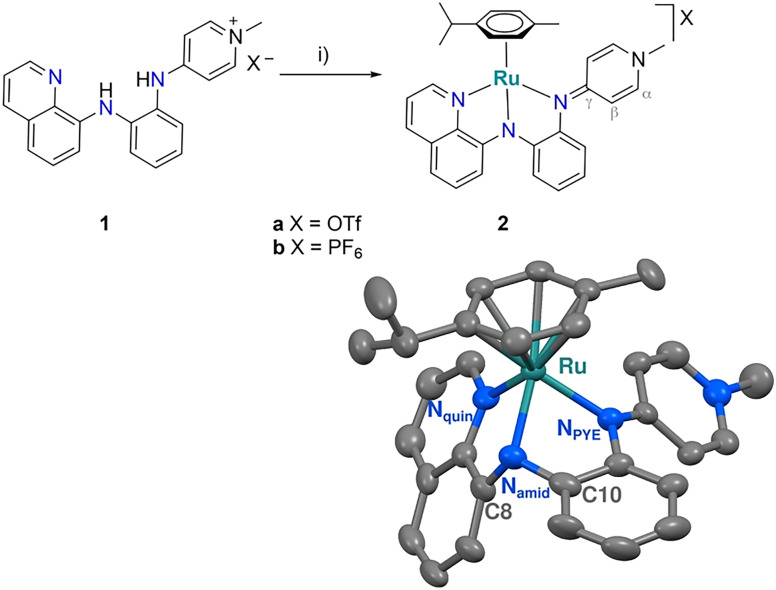
Synthesis of the NNN Ru complexes 2a and 2b. i) [Ru(cym)Cl_2_]_2_, Na_2_CO_3_, CH_3_CN, r.t.; crystal structure of complex 2a (ellipsoids at 50% probability level. Hydrogen atoms and OTf^−^ anion omitted for clarity). Selected bond lengths (Å) and angles (deg): Ru–N_quin_ 2.089(6), Ru–N_amid_ 2.068(5), Ru–N_PYE_ 2.123(6), C8–N_amid_–C10 115.7(6), Ru1–N_amid_–C10 108.3(5), Ru–N_amid_–C8 114.4(5), N_quin_–Ru–N_amid_ 78.6(2), N_amid_–Ru–N_PYE_ 75.7(2), N_quin_–Ru–N_PYE_ 93.3(2).

The potentially *N*,*N*,*N*-tridentate coordinating ligand precursor 1 is conveniently accessible from 8-aminoquinoline in three simple steps.^36^ Reaction of 1 with [Ru(cym)Cl_2_]_2_ in the presence of Na_2_CO_3_ afforded complex 2a as a red solid that was purified by filtration over a short pad of basic Al_2_O_3_ (Scheme 1). Complex 2b with a PF_6_^−^ rather than an OTf^−^ anion was prepared by first generating the neutral ligand from 1 in aqueous KOH solution and extraction into CH_2_Cl_2_, followed by metalation with [Ru(cym)Cl_2_]_2_ in the presence of NaPF_6_ and Na_2_CO_3_. Both complexes 2a and 2b are air- and moisture-stable, however they are sensitive to light. Therefore, synthesis and purification were carried out under exclusion of light, and the complexes were stored in the dark.

Complex formation was indicated in HR-MS analysis by the diagnostic *m*/*z* signals at 561.1575 (561.1587 calculated for [2a–OTf]^+^). In ^1^H NMR spectroscopy, complexation led to the disappearance of the two NH resonances of the ligand precursor 1 at *δ*_H_ = 10.12 and 8.63, and to desymmetrization of the *i*Pr group of the cymene ligand into two doublets at *δ*_H_ = 0.89 and 0.86 for the CH_3_ protons. Moreover, the quinoline CH resonances underwent significant downfield shifts (*e.g.* C_quin_H from *δ*_H_ = 9.03 in 1 to *δ*_H_ = 9.43 in 2a), in agreement with ruthenium coordination of the quinoline nitrogen. Likewise, coordination of the PYE site was inferred from the characteristic upfield shift of the N_pyr_–CH_3_ resonance from *δ*_H_ = 3.86 to 3.48 upon ruthenation. Collectively, these data strongly support a tridentate coordination of the ligand to ruthenium with all nitrogens deprotonated. The ^1^H NMR spectrum of complex 2b is identical to that of 2a, yet ^19^F NMR spectroscopy shows the diagnostic doublet at *δ*_F_ = −72.94 for the PF_6_^−^ anion, while 2a features a singlet at *δ*_F_ = −79.31 due to the OTf^−^ anion. The indistinguishable ^1^H NMR spectra of 2a and 2b indicate that the counterions are not strongly interacting with the complex cation in CD_3_CN solution.

X-ray diffraction analysis of single crystals of 2a, grown by Et_2_O/CH_2_Cl_2_ vapor diffusion, unambiguously confirmed the proposed *N*,*N*,*N*-tridentate coordination mode of the substituted PYE ligand to the Ru(cym) fragment in a facial mode (Scheme 1). The PYE heterocycle is slightly twisted out of the plane of the central phenylene ring by 36.22°. The C–C and C–N bond distances in the PYE heterocycle are consistent with considerable double bond localization in the C_α_–C_β_ bond (average 1.36(1) Å), while the C_β_–C_γ_ bonds are significantly longer (average 1.42(1) Å), indicative of a predominantly quinoidal configuration of the PYE unit in the solid state with a neutral N_PYE_ coordinated to ruthenium. The 360° sum of the bond angles around N_PYE_ also suggest sp^2^ hybridization, identical to N_quin_. In contrast, the central amide shows considerable sp^3^ character with bond angles adding up to just 338°, pointing to a lone pair at N_amid_. Notably, the NNN-tridentate coordination imparts a three-legged piano-stool geometry around Ru that is markedly distorted towards a four-legged geometry with a vacant site opposite to N_amid_ (Fig. S12†).

**Fig. 1:**
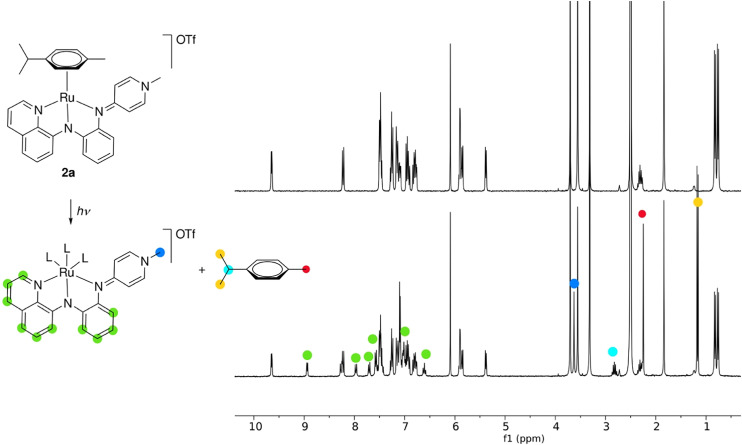
^1^H NMR spectra of 2a in DMSO-*d*_6_ under N_2_ and exclusion of light (top), and after exposure to sunlight for 1 h (bottom).

When exposed to light, solutions of complex 2 gradually darkened, irrespective of the counterion (PF_6_^−^ or OTf^−^) or the solvent (DMSO, acetone, CH_2_Cl_2_, MeCN). When kept under otherwise inert conditions, light-induced decomposition was indicated in ^1^H NMR spectroscopy by the appearance of resonances characteristic for free cymene, *e.g.*, a doublet at *δ*_H_ = 1.17 in DMSO-*d*_6_ (*cf.* two doublets at *δ*_H_ = 0.82 and 0.76 for 2a; Fig. 1). Simultaneously a new set of signals emerged in the aromatic region for the *N*,*N*,*N*-tridentate ligand that are distinct from the ligand precursor. We therefore tentatively attribute these signals to the formation of a *solvento* complex [Ru(NNN)(DMSO)_3_]^+^. When complex 2 was exposed to light in the presence of oxygen, again decoordination of cymene was inferred. In addition, a new species emerged with broad and poorly resolved signals, suggesting metal oxidation and the formation of a paramagnetic Ru(iii) species. The same behavior was observed when air was introduced into a solution of the putative solvent complex [Ru(NNN)(DMSO)_3_]^+^. ESI mass spectrometry of solutions of complex 2a after exposure to light and air revealed an *m*/*z* signal at 459.0376 (459.0395 calculated for [2a + O_2_–cym–OTf]^+^) consistent with a higher-valent ruthenium dioxo or oxyl species that preserves the NNN ligand. These data and the absence of any detectable ligand precursor suggest that the NNN-ligand remains coordinated to the ruthenium during the various ligand exchange and metal oxidation processes. In contrast, when complex 2 was protected from light, no loss of cymene ligand was observed for weeks, even when the solutions were aerated, indicating that cymene dissociation is key for metal oxidation processes. For application, any oxidized species is conveniently removed by filtration of the complex over a short pad of basic Al_2_O_3_ (acetone/CH_2_Cl_2_ 1 : 2).

**Fig. 2:**
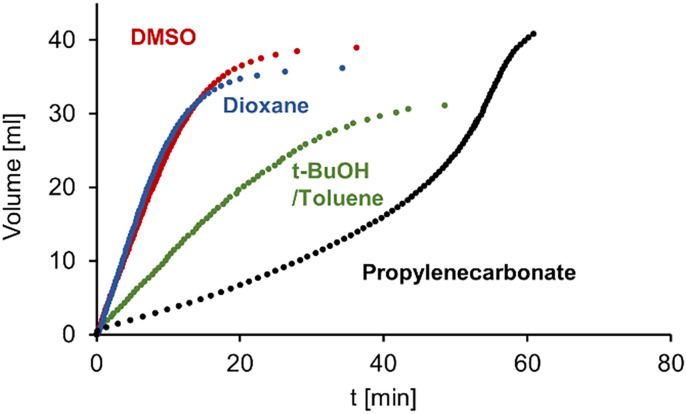
Time-dependent gas evolution profiles for FA dehydrogenation with 2a in different solvents. Reaction conditions: FA (40 μL), [Ru] (0.2 mol%), solvent (1.2 mL), 80 °C under N_2_. Representative single runs are shown but catalytic runs were performed at least twice to ensure reproducibility (deviation <5%). The profile in H_2_O is omitted as no activity was observed.

Complexes 2a and 2b are catalytically active in the dehydrogenation of FA. Initial reactions were performed by adding FA to a solution of 2a at 80 °C and subsequent monitoring of the amounts of evolved gas (Fig. 2). Several solvents were tested that were previously used in FA dehydrogenation catalysis, including dioxane, DMSO, water, propylene carbonate, and a mixture of *t*BuOH and toluene (1 : 1 v/v; Table 1).^12,26,33,37,38^ In water the complex was completely inactive (entry 1). Highest catalytic activities were observed in DMSO and in dioxane, with initial turnover frequencies (TOFs) around 2000 h^−1^ (entries 2,3). A mixture of toluene and *t*BuOH suppressed activity considerably (TOF = 710 h^−1^; entry 4). Interestingly, using propylene carbonate as solvent completely changed the reaction profile (Fig. 2). While the initial activity was low (TOF = 200 h^−1^), it gradually increased over the course of about one hour to reach an appreciable TOF_max_ = 1300 h^−1^ (entry 5). A similar unusual gas evolution profile has been reported previously with carbonate solvents.^38^ It may be rationalized by either a slow catalyst activation in propylene carbonate, by initial hydrogenation of the solvent and formation of *n*PrOH as a co-solvent, or by a gradual pH increase upon formic acid consumption to an optimal range with highest catalyst performance. To probe these scenarios, another batch of FA was added after completion of the initial run. These experiments resulted in a similar gas evolution profile with gradually increasing catalytic activity, albeit at lower overall rate. This behavior is consistent with a pH-dependent catalyst performance rather than solvent hydrogenation or a long induction due to catalyst activation. Irrespective of its exact role, propylenecarbonate lowers the activity of 2a considerably when compared to dioxane or DMSO. The latter solvent is preferable over dioxane as it is non-toxic, more sustainable,^39^ features a low vapor pressure, and a high boiling point. Even though the exact role of the solvent is not clear and may be multifaceted (*e.g.* influencing the p*K*_a_ of the complex, or catalytic intermediates, polarity to stabilize transition states), the observed dependence might speculatively hint also to weak coordination to the ruthenium center.

**Table 1:** FA dehydrogenation catalysis with 2a in different solvents^a^

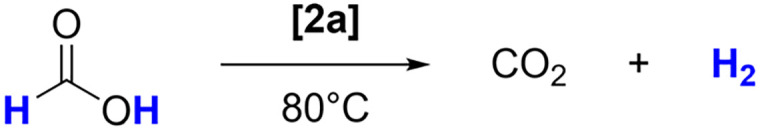				
Entry	Solvent	mol% 2a	TOF_max_^b^	TON^b^
1	H_2_O	0.2	—	0
2	Dioxane	0.2	2000	380
3	DMSO	0.2	2000	420
4	*t*-BuOH/toluene	0.2	710	310
5	Propylene carbonate	0.2	1300	440
6	DMSO	0.02	9200	1400
7	DMSO	0.002	1300	2200

a Reaction conditions: FA (40 μl), complex 2a, solvent (1.2 mL), 80 °C under N_2_.

b TON and TOF determined based on evolved gas volume, see ESI† for details (accuracy at ±5%).

**Fig. 3:**
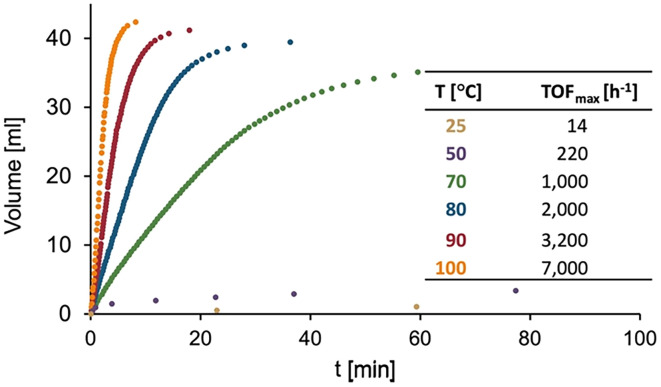
Time-gas evolution profiles for FA dehydrogenation with 2a at different temperatures (accuracy of TOF ± 5%). Reaction conditions: FA (40 μL), [Ru] (0.2 mol%), DMSO (1.2 mL), under N_2_.

Complexes 2a and 2b show identical conversion profiles and catalytic performance in DMSO, indicating no significant influence of the counterion on the catalytic cycle. Performing FA dehydrogenation at higher temperatures notably increases TOFs from 2000 h^−1^ at 80 °C to 3200 h^−1^ at 90 °C and 7000 h^−1^ at 100 °C, while lowering the temperature has the expected opposite effect (Fig. 3). At room temperature, the catalysis is essentially stalled with TOF <20 h^−1^. A tenfold decrease in pre-catalyst loading from 0.2 mol% to 0.02 mol% increased the TOF to 9200 h^−1^ at 80 °C (Table 1, entry 6), though a further lowering to 0.002 mol% significantly impeded catalyst turnover (TOF = 1300 h^−1^; entry 7), presumably because impurities in the solvent and/or FA become more significant with these low catalyst loadings. Based on these turnover frequencies, complex 2a is showing outstanding activity and outperforms most other ruthenium-based catalysts (Table 2),^27,40–44^*e.g.* Milstein's system features a TOF around 3000 h^−1^ (in neat FA)^27^ and Fischmeister's complex a TOF of 230 h^−1^ (in DMSO), though it does not reach the performance of Grützmacher's Ru(trop_2_dad) catalyst with TOFs up to 24 000 h^−1^.^42^ While iridium catalysts show generally an even higher activity with TOFs that are up to 2 orders of magnitude larger,^26^ ruthenium is about 100 times less scarce and about 10 times cheaper. Notably, under dilute catalyst conditions, the maximum turnover number (TON) of 2a was just 2200, which is a significant limitation in comparison to the Milstein catalyst with 1,7 M TON.

**Table 2:** Selected ruthenium complexes in base-free formic acid dehydrogenation

Entry	Complex	*T*/°C	TOF_max_	TON	Ref.
1	Ru(cym)(NNN) 2a	80	9200	2200	This work
2	RuH(CO)(PNP)	90	3000	1 700 000	27
3	[RuCl(P^arene–O)]_2_	90	200	170	40
4	[RuCl(cym)(bis-imid)]_2_	90	750	2000	41
5	RuH(trop_2_dad)	90	24 000	8100	42
6	RuH(PNP)(CO)	50	2400	95 000	43
6	RuH_2_(H_2_)(PPh_3_)_3_	60	2400	2000	44
7	RuH_2_(PPh_3_)_4_	60	12 000	19 000	44

Arrhenius and Eyring analyses of the initial rates of these temperature-dependent catalytic runs revealed an activation energy *E*_a_ = 68 ± 1 kJ mol^−1^, an enthalpy of activation Δ*H*^‡^ = 65 ± 1 kJ mol^−1^ and an entropy of activation Δ*S*^‡^ = −177 ± 3 J K^−1^ (Fig. S19 and S20†). The highly negative Δ*S*^‡^ suggests an associative process in the turnover limiting step, which might entail, for example, the interaction between a putative ruthenium hydride and FA, or adduct formation of the ruthenium complex and *in situ* formed formate.

Catalytic dehydrogenation with complex 2a is accompanied by distinct color changes of the reaction solution. The initially red complex solution turns immediately purple upon addition of FA at elevated temperatures and then gradually changes to orange. Monitoring the catalytic reaction by *in situ* UV-vis spectroscopy indicates that the color change from the red complex solution (*λ*_max_ = 500 nm) to the purple solution (*λ*_max_ = 550 nm) is essentially instantaneous (<1 s) and coincides with the start of gas evolution, *i.e.* catalytic activity (Fig. S23 and S24†). After about 8 min, a new band starts to emerge (*λ*_max_ = 445 nm). The gas evolution rate decreases as this band is growing, and the corresponding species is persistent at the end of the reaction when gas evolution ceased, indicating that the orange Ru species is catalytically inactive.

**Scheme 2:**
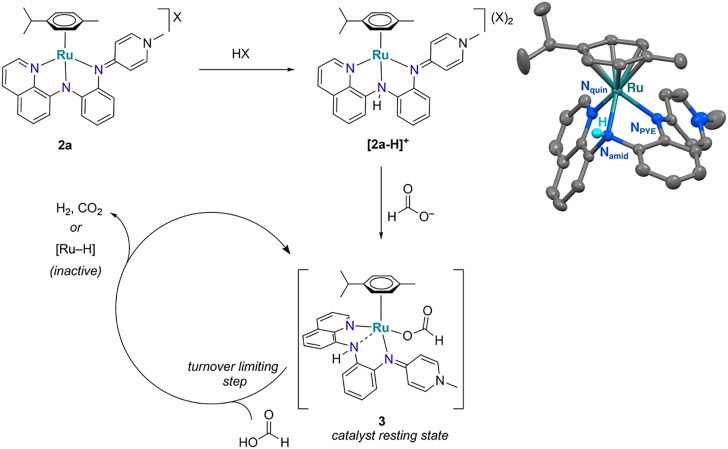
Suggested pathway for the activation of complex 2 for catalytic FA dehydrogenation and thermal ellipsoid plot of **[**2a**–H]^+^** (50% ellipsoids, the two PF_6_^−^ counteranions, all carbon-bound hydrogen atoms, and co-crystallized CH_2_Cl_2_ molecule omitted for clarity).

Further details unveiled upon lowering the temperature of the catalytic reaction to 30 °C. At this temperature, formation of the active purple species is not instantaneous anymore, and instead, an immediate color change from red to yellow was noted upon formic acid addition, followed by a gradual color change to purple. The yellow species is characterized by the disappearance of the absorbance at 500 nm from complex 2a, with only intra-ligand charge transfer bands present in the UV region (Fig. S21†). No gas evolution was recorded at this stage. The evolution of the yellow species, has been attributed to protonation of the *N*,*N*,*N*-ligand at its central nitrogen, which is supported by reactions performed with catalytically innocent acids such as TFA or HOTf (TFA = CF_3_COOH, HOTf = CF_3_SO_3_H). Thus, when treating a DMSO solution of the ruthenium complex 2a with TFA (1 eq.) instead of formic acid induced the same instantaneous color change from red to yellow and yielded **[**2a**–H]^+^** (Scheme 2). The ^1^H NMR spectrum of **[**2a**–H]^+^** revealed a new resonance at *δ*_H_ = 11.8, consistent with an acidic NH functionality (Fig. S27†). Protonation of the central Ar–N–Ar nitrogen is deduced based on only relatively small chemical shifts of the heterocyclic protons. It agrees with the considerable pyramidalization and sp^3^ hybridization of the N_amid_ as evidenced by the molecular structure of 2a, and was ultimately confirmed by a single-crystal X-ray diffraction of the PF_6_^−^ salt of **[**2a**–H]^+^** (Scheme 2, Fig. S12†). While the global structure of **[**2a**–H]^+^** is almost identical to that of 2a, all bonds to N_amid_ are significantly elongated (*e.g.* Ru–N_amid_ is 2.068(5) Å in 2a*vs.* 2.132(3) Å in **[**2a**–H]^+^**) and thus corroborate protonation of this nitrogen. The marked deshielding of the NH resonance (*cf. δ*_H_ = 8.4 in 1) suggests that also in solution, the nitrogen remains coordinated to ruthenium upon protonation. The moderate downfield shift of the aromatic signals, *e.g.* the *ortho* CH_quin_ from *δ*_H_ = 9.64 to 9.96, is in agreement with increased N_quin_-to-Ru charge transfer and points to some compensation of the reduced amine basicity upon protonation. The same spectroscopic changes were noted when treating 2a with 10 eq. HOTf, indicating selective protonation of only one nitrogen (Fig. S10†). Addition of Na_2_CO_3_ fully reverses the color change back to red and restores the ^1^H NMR spectrum of 2a, identifying protonation as a completely reversible process.

The subsequent appearance of the purple color features an isosbestic point at 440 nm and is again accompanied by gas evolution, indicating a direct conversion of **[**2a**–H]^+^** into the catalytically competent species. Within the inital 7 min *λ*_max_ is located at 550 nm as observed at 80 °C, and gas evolution is linear. The absorption then slowly decreases and the maximum shifts to 530 nm during the subsequent 60 min, and gas evolution gradually ceases (Fig. S22†). Hence the absorption shift relates to the formation of a catalytically inactive species. In agreement, NMR signals due to free *p*-cymene became visible at this stage of gas evolution, thus indicating a pathway for catalyst deactivation.

Structural information about the purple catalytically active species has been gathered from the reaction of complex 2a with 180 eq. FA in DMSO-*d*_6_ to mimic catalytic conditions (Fig. S26–S29†). Addition of FA induces again an instantaneous color change to yellow and NMR spectroscopy shows the formation of **[**2a**–H]^+^**. In addition, a new species starts to emerge which features signals for the *N*,*N*,*N*-ligand and the cymene that are only slightly shifted with respect to **[**2a**–H]^+^**, suggesting robust coordination of both ligands. The evolution of this component, tentatively attributed to the formic acid complex 3 (Scheme 2), increases gradually and is persistent for over 2 h, during which gas evolution is essentially linear under these conditions (Fig. S26 and S30†). Of note, the NNN-ligand is likely protonated as indicated by a strongly deshielded singlet at *δ*_H_ = 10.28 ppm, which is absent when the reaction is performed with DCOOD instead of HCOOH (Fig. S29†). Therefore, N_amid_ is coordinating only weakly, if at all, to the ruthenium center in complex 3. While the tridentate chelation of the NNN-ligand principally might provide space for a small ligand to bind to the ruthenium center in a distorted square-pyramidal geometry (Fig. S12†), hemilability of the central nitrogen is more likely to accommodate the formate and is not unprecedented.^36^

In the high-concentration regime of the NMR experiment, additionally, trace amounts of a hydride species are present at the initial stages of the reaction, with a diagnostic signal at *δ*_H_ = −6.14 and corresponding resonances in the cymene and NNN ligand regions (Fig. S26†). After about 20 min, this hydride species is fully consumed, though gas evolution continues for another two hours in an almost linear regime, suggesting that this hydride complex is not related to the catalytically active species. This hydride side product is also visible in mixtures resulting from stoichiometric reactions of 2a with 1 eq. formic acid, or when treating **[**2a**–H]^+^** with stoichiometric quantities of lithium formate (Fig. S25†).

The relevance of these room temperature studies was validated by performing a formic acid dehydrogenation experiment at 80 °C for just 1 min followed by immediate cooling. The corresponding ^1^H NMR spectrum is essentially identical to that from room temperature reactions after 35 min (Fig. S31 and S32†) and also supports the variable temperature UV-vis data (*vide supra*). When FA is almost fully consumed additional hydridic resonances at *δ*_H_ = −11.06 and −14.3 are observed, indicating that upon consumption of all formic acid, either cymene is lost or the tridentate coordination mode of the NNN ligand is altered.

Overall, these spectroscopic data suggest fast N-protonation as the initial catalyst activation step. Proton transfer to the ruthenium-bound amide nitrogen is pH controlled and fast in the presence of excess FA or a strong acid. This protonation is assumed to weaken the Ru–N_amid_ bond, and thus allow for formate coordination. This purple species 3 is considered the catalyst resting state. While it is tempting to propose a β-hydrogen elimination from this resting state to form a hydride species, such a turnover-limiting step would imply a highly positive entropy of activation because of the concomitant release of CO_2_, which is incompatible with the largely negative value of Δ*S*^‡^ (*vide supra*). We therefore propose the formation of a highly ordered adduct with a second equivalent of formic acid to facilitate the hydride formation (Scheme 2). Note that due to the distal location of the acidic proton at N_amid_ from the putative ruthenium-bound hydride, intramolecular elimination of H_2_ seems much less likely compared to protonation of the hydride by extraneous formic acid. In this latter scenario, N_amid_ protonation is essential for opening up a coordination site at the ruthenium center during catalyst activation, but not for cooperative formic acid activation or H_2_ release.

## Conclusion

We disclose here a ruthenium complex containing an easily accessible NNN ligand system for efficient formic acid dehydrogenation. The catalytic system shows very high catalytic activity with turnover frequencies that surpass most other additive-free ruthenium catalyst for formic acid dehydrogenation. Mechanistic investigations indicate a purple phase as catalytically active species, which is defined by a ruthenium system that still comprises the cymene and the NNN ligand, though the latter presumably only in bidentate coordination mode. While the system is highly active, its longevity is mediocre. This limitation may be addressed by deliberate ligand modifications, which are straightforward because of the convenient and modular ligand synthesis, and possibly also by catalyst immobilization, which has previously shown to enhance the catalyst longevity considerably.^29^

## Conflicts of interest

The authors declare no competing financial interests.

## Supplementary Material

CY-013-D3CY00512G-s001

CY-013-D3CY00512G-s002
