# Supplementary material for: Efficient additive-free formic acid dehydrogenation with a NNN–ruthenium complex
Source: Catal Sci Technol. 2023 Jul 17;13(19):5625–31. doi: 10.1039/d3cy00512g (PMC10544809; doi:10.1039/d3cy00512g)
Supplement: CY-013-D3CY00512G-s001 [file CY-013-D3CY00512G-s001.pdf]

Electronic Supplementary Material (ESI) for Catalysis Science & Technology.
This journal is © The Royal Society of Chemistry 2023

Electronic Supplementary Information

belonging to

Efficient additive-free formic acid dehydrogenation with a NNN-ruthenium complex

*Pascal Knörr, Nicolas Lentz, Martin Albrecht**

Department of Chemistry, Biochemistry & Pharmaceutical Sciences, University of Bern, Freiestrasse 3, 3012 Bern (Switzerland).

Table of Contents

1. Synthetic procedures S2
2. NMR Spectra of all new compounds S5
3. Reversible Acid-Base reaction S10
4. Single crystal X-ray diffraction analysis S12
5. Catalytic data S13
6. Mechanistic data S18
7. Crystallographic details S30
8. References S31

1. Synthetic procedures

General

Ruthenium complexes were synthesized under exclusion of light. Ligand precursor **1a** was synthesized according to a published procedure.[1] All other reagents were commercially available and used as received unless specified. NMR spectra were recorded at 25 °C on Bruker spectrometers operating at 300 or 400 MHz (^{1}H NMR), 282 or 376MHz (^{19}F NMR), and 75 or 100 MHz (^{13}C{^{1}H} NMR), respectively. Chemical shifts (δ in ppm, coupling constants *J* in Hz) were referenced to residual solvent signals (^{1}H,^{13}C). Assignments are based on homo- and hetero nuclear shift correlation spectroscopy. High resolution mass spectrometry was carried out by the DCBP mass spectrometry group at the University of Bern with a Thermo Scientific LTQ Orbitrap XL (ESI-TOF). Elemental analyses were performed on a Thermo Scientific Flash 2000 CHNS-O elemental analyzer by the DCBP Microanalytic Laboratory.

Synthesis of complex 2a

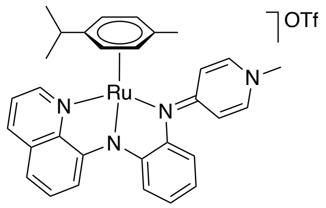

Under nitrogen atmosphere and under exclusion of light, [Ru(*p*-cymene)Cl_2]$_2$ (128.5 mg, 0.21 mmol), ligand precursor **1a** (200 mg, 0.42 mmol) and Na_2CO_3 (444 mg, 4.2 mmol) were stirred in degassed CH_3CN (11 mL) for 16 h. The crude mixture was filtered, and all volatiles were removed under reduced pressure. The residue was dissolved in CH_2Cl_2 and filtered over a short pad of basic Al_2O_3. The complex was eluted with a mixture of CH_2Cl_2 and acetone (2:1; 60 mL) as a red solution. All volatiles were evaporated under reduced pressure and the solid redissolved in a minimal amount of CH_2Cl_2. Et_2O was then added until a red solid precipitated. The supernatant was removed and the solid washed with Et_2O (2 × 10 mL) and pentane (2 × 10 mL) to yield complex **2a** as a red solid (190 mg, 64%). Slow diffusion of Et_2O into a solution of complex **2a** in CH_2Cl_2 gave crystals suitable for X-ray diffraction analysis.

^{1}H NMR (300 MHz, CD_3CN) δ 9.44 (dd, $^3J_{HH}$ = 5.1, $^4J_{HH}$ = 1.3 Hz, 1H, H_{Quin}), 8.14 (dd, $^3J_{HH}$ = 8.4, $^4J_{HH}$ = 1.3 Hz, 1H, H_{Quin}), 7.48 (dd, $^3J_{HH}$ = 7.6, $^4J_{HH}$ = 1.4 Hz, 1H, H_{Ar}), 7.38 (dd, $^3J_{HH}$ = 8.4, 5.1 Hz, 1H, H_{Quin}), 7.32–7.23 (m, 2H, $H_{PYE\ \alpha}$, H_{Quin}), 7.18 (dd, J_{HH} = 3.5, 1.3 Hz, 1H, H_{Ar}), 7.16 (dd, J_{HH} = 3.3, 1.3 Hz, 1H, H_{Ar}), 7.02 (dd, J_{HH} = 7.6, 3.1 Hz, 1H, H_{Ar}), 7.00–6.92 (m, 3H, $H_{PYE\ \alpha}$, 2 H_{Ar}), 6.83 (td, J_{HH} = 7.6, 1.4 Hz, 1H, H_{Ar}), 6.78 (dd, J_{HH} = 7.7, 3.1 Hz, 1H, H_{Ar}), 5.78–5.71 (m, 2H, H_{cym}), 5.69–5.62, 5.18–5.12 (2 × m, 1H, H_{cym}), 3.48 (s, 3H, N–CH_3), 2.37 (septet, $^3J_{HH}$ = 6.9 Hz, 1H, CHMe$_2$), 1.91 (s, 3H, cym–CH_3), 0.87, 0.83 (2 × d, $^3J_{HH}$ = 6.9 Hz, 3H, (CH_3)$_2$CH). ^{13}C NMR (101 MHz, CD_3CN) δ 160.92, 160.25, 159.64 (3 × C_{Ar}), 154.68, 154.62(1 ×CH_{Quin}, 1 × C_{Ar}), 148.23, 141.47, 140.86 (3 × C_{Ar}), 138.24(CH_{Quin}), 131.01, 129.98, 125.52, 124.50 (4 × C_{Ar}), 123.20 (CH_{Quin}), 122.78, 120.15, 119.17, 116.57, 114.62, 109.51 (6 × C_{Ar}), 103.18, 102.58 ((C_{cym}–*i*Pr, C_{cym}–Me), 88.16, 87.93, 86.54, 84.72 (4 × C_{cym}–H), 43.84 (N–CH_3), 31.31 (CHMe$_2$)), 22.77, 21.73(2 × CH(CH_3)$_2$), 18.54 (cym–CH_3). ^{19}F NMR (376 MHz, CD_3CN) δ -79.31 (OTf). HR-MS (m/z): Calculated for [M–OTf] = 561.1587 found 561.1575. Anal. Calc. (%) for $C_{32}H_{31}F_3N_4O_3RuS$: C 54.15, H 4.40, N 7.89; found C 53.73, H 4.35, N 7.63.

Synthesis of complex 2b

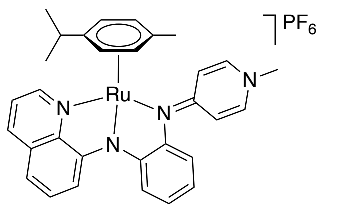

Ligand precursor **1a** (400 mg, 0.84 mmol), was stirred in a biphasic mixture of aqueous KOH (1 M, 10 mL) and CH_2Cl_2 (20 mL) for 15 min. The CH_2Cl_2 phase was separated and washed with 0.1 M KOH solution (20 mL) followed by water (20 mL). The organic phase was dried over Mg_2SO_4, filtered, and all volatiles were removed under reduced pressure. The resulting oil was layered with pentane for 1 h to afford a solid, which was washed with pentane (2 × 20 mL) and dried in vacuo to yield a yellow waxy solid (272 mg, 99%). This solid was directly used for further synthesis without full characterisation.

^{1}H NMR (300 MHz, CD3CN) δ 8.81 – 8.71 (m, 2H), 8.20 (dd, $^3J_{HH}$ = 8.3, $^4J_{HH}$ = 1.7 Hz, 1H), 7.67 (dt, $^3J_{HH}$ = 7.7, $^4J_{HH}$ = 1.0 Hz, 1H), 7.60 (dd, $^3J_{HH}$ = 7.8, $^4J_{HH}$ = 1.2 Hz, 1H), 7.54 – 7.40 (m, 2H), 7.25 (dd, $^3J_{HH}$ = 8.2, $^4J_{HH}$ = 1.2 Hz, 1H), 7.10 – 6.88 (m, 4H), 6.45 – 6.00 (m, 2H), 3.40 (s, 3H).

Under nitrogen atmosphere and exclusion of light, the waxy solid (80 mg, 0.25 mmol), [Ru(*p*-cymene)Cl_2]$_2$ (75 mg, 0.13 mmol), $NaPF_6$ (41 mg, 0.25 mmol) and Na_2CO_3 (130 mg, 1.2 mmol) were stirred in degassed CH_3CN (5 mL) for 40 h. The crude mixture was filtered, and all volatiles were removed under reduced pressure. The residue was dissolved in CH_2Cl_2 and filtered over a short pad of basic Al_2O_3. The complex was eluted with a mixture of CH_2Cl_2 and acetone (2:1; 60 mL) as a red solution. All volatiles were evaporated under reduced pressure and the solid redissolved in a minimal amount of CH_3CN. Then, Et_2O was added until a red solid precipitated. The supernatant was removed and the solid washed with Et_2O (2 × 10 mL) and pentane (2 × 10 mL) to yield complex **2b** as a red solid (135 mg, 78%).

^{1}H NMR and ^{13}C NMR spectra are identical to those of **2a.** ^{19}F NMR (376 MHz, CD_3CN) δ -72.94 (d, J = 706.5 Hz, PF_6). HR-MS (m/z): Calculated for $[M–PF_6]^+$ = 561.1581 found 561.1579.Anal. Calc. (%) for $C_{31}H_{31}F_6N_4PRu$: C 52,77, H 4.43, N 7.94 found C 52.72, H 4.41, N 7.79.

Synthesis of complex [2a–H]$^+$

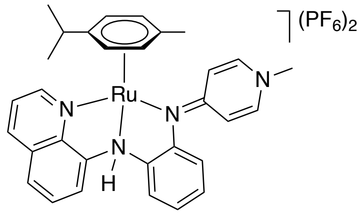

2a (297.9 mg, 0.42 μmol) was dissolved in CH_3CN (10 mL). HOTf (0.12 mL, 1.26 mmol) was added until colour changed from inital red to yellow. Aqueous saturated NH_4PF_6 solution (3 mL) and water (40 mL) were added, leading to precipitation of a yellow solid. The solid was filtered of, washed with water (40 mL) and dried under reduced pressure. The solid was dissolved in CH_2CH_2 (10 mL) and stirred for 10 min over an excess of $NaPF_6$ (400 mg). The solution was filtered and concentrated under reduced pressure. Et_2O was added (50 mL), leading to a yellow precipitate which was washed with Et_2O (2 × 20 mL). All volatiles were removed under reduced pressure to afford **[2a-H][2PF$_6$]** as a yellow solid (215 mg, 60%)

^{1}H NMR (400 MHz, CD_3CN) δ 10.44 (s, 1H), 9.65 (dd, $^3J_{HH}$ = 5.2, $^4J_{HH}$ = 1.4 Hz, 1H, NH), 8.53 (dd, $^3J_{HH}$ = 8.5, $^4J_{HH}$ = 1.3 Hz, 1H, H_{Quin}), 8.02 (dt, $^3J_{HH}$ = 7.6, $^4J_{HH}$ = 1.0 Hz, 1H, H_{Quin}), 7.96 (dd, $^3J_{HH}$ = 8.3, $^3J_{HH}$ = 1.1 Hz, 1H, H_{Quin}), 7.88 (dd, $^3J_{HH}$ = 7.8, $^4J_{HH}$ = 1.5 Hz, 1H, H_{Ph}), 7.73 (t, $^3J_{HH}$ = 7.9 Hz, 1H, H_{Quin}), 7.66 (dd, $^3J_{HH}$ = 8.4, $^3J_{HH}$ = 5.2 Hz, 1H, H_{Quin}), 7.35 (dd, $^3J_{HH}$ = 7.9, $^4J_{HH}$ = 1.4 Hz, 3H, H_{Ph}), 7.45 – 7.22 (broad m, 2H, H_{PYE}), 7.16 (td, $^3J_{HH}$ = 7.7, $^4J_{HH}$ = 1.5 Hz, 1H, H_{Ph}), 7.09 (td, $^3J_{HH}$ = 7.6, $^3J_{HH}$ = 1.4 Hz, 1H, H_{Ph}), 6.79 (s, 2H, H_{PYE}), 6.24 (dd, $^3J_{HH}$ = 6.1, $^4J_{HH}$ = 1.3 Hz, 1H, H_{cym}), 6.10 (dd, $^3J_{HH}$ = 6.1, $^4J_{HH}$ = 1.3 Hz, 1H, H_{cym}), 6.01 (dd, $^3J_{HH}$ = 6.6, $^3J_{HH}$ = 1.0 Hz, 1H, H_{cym}), 5.86 (dd, $^3J_{HH}$ =

6.1, $^3J_{HH}$ = 1.3 Hz, 1H, H_{cym}), 3.60 (s, 3H), 2.48 (sept, $^3J_{HH}$ = 6.9 Hz, 1H, CHMe$_2$), 1.91 (s, 3H, cym–CH_3), 0.90 (d, $^3J_{HH}$ = 6.9 Hz, 3H, (CH_3)$_2$CH), 0.86 (d, $^3J_{HH}$ = 6.9 Hz, 3H, (CH_3)$_2$CH). ^{13}C NMR (101 MHz, CD$_3$CN) δ 162.14 (C$_{Ar}$), 158.84 (CH$_{Quin}$), 149.54, 147.51, 145.61, 142.93, 142.47 (5 × C$_{Ar}$), 140.89 (CH$_{Quin}$), 130.32, 130.13, 129.84, 129.82, 129.01, 125.02, 123.45, 122.69 , 106.96, 103.32 (10 × C$_{Ar}$), 86.62, 85.65, 85.17, 84.73 (4 × C$_{cym}$–H), 44.60 (N–CH$_3$), 31.37 (CHMe$_2$)), 22.20, 21.86 (2 × CH(CH$_3$)$_2$), 18.14. Anal. Calc. (%) for $C_{31}H_{32}F_{12}N_4P_2Ru$: C 43.72, H 3.79, N 6.58; found C 43.69, H 3.93, N 6.32.

In situ protonation with excess OTf

Complex **2a** (4 mg, 5.64 µmol) was dissolved in DMSO-d$_6$ and HOTf (ca. 5 µL, 56 µmol) was added. Upon addition the red solution immediately turned yellow.

^{1}H NMR (300 MHz, DMSO-d_6) δ 12.04 (s, 1H), 9.98 (d, $^3J_{HH}$ = 5.1 Hz, 1H), 8.70 (d, $^3J_{HH}$ = 8.3 Hz, 1H), 8.20 (d, $^3J_{HH}$ = 7.6 Hz, 1H), 8.06 (d, $^3J_{HH}$ = 8.1 Hz, 1H), 7.96 (dd, $^3J_{HH}$ = 7.6, $^4J_{HH}$ = 1.6 Hz, 1H), 7.84 – 7.73 (m, 2H), 7.55 (s, 2H), 7.35 (dd, $^3J_{HH}$ = 7.9, $^4J_{HH}$ = 1.6 Hz, 1H), 7.21 – 7.04 (m, 2H), 6.86 (s, 2H), 6.33 (t, $^3J_{HH}$ = 6.5 Hz, 2H), 6.15 – 6.07 (m, 2H), 3.67 (s, 3H), 2.48 – 2.38 (m, 1H), 1.88 (s, 3H), 0.85 (d, $^3J_{HH}$ = 6.9 Hz, 3H), 0.81 (d, $^3J_{HH}$ = 6.8 Hz, 3H).

Reversible deprotonation

Na_2CO_3 (ca. 8 mg, 75 µmol) was added and the suspension mixed. The color of the solution changed back from yellow to red. The ^{1}H NMR spectrum reverted to that of **2a**.

Light sensitivity of complexes 2a and 2b

In an NMR tube DMSO-d$_6$ solutions of **2a/2b** (4 mg in 0.6 mL) were kept in the dark and respective ^{1}H NMR spectras were recorded. The tubes were then taped to a sunfacing window and after regular time intervals ^{1}H NMR spectras were recorded to monitor the decomposition. The experiments were performed both under inert atmosphere and in air.

2. NMR Spectra of new Compounds

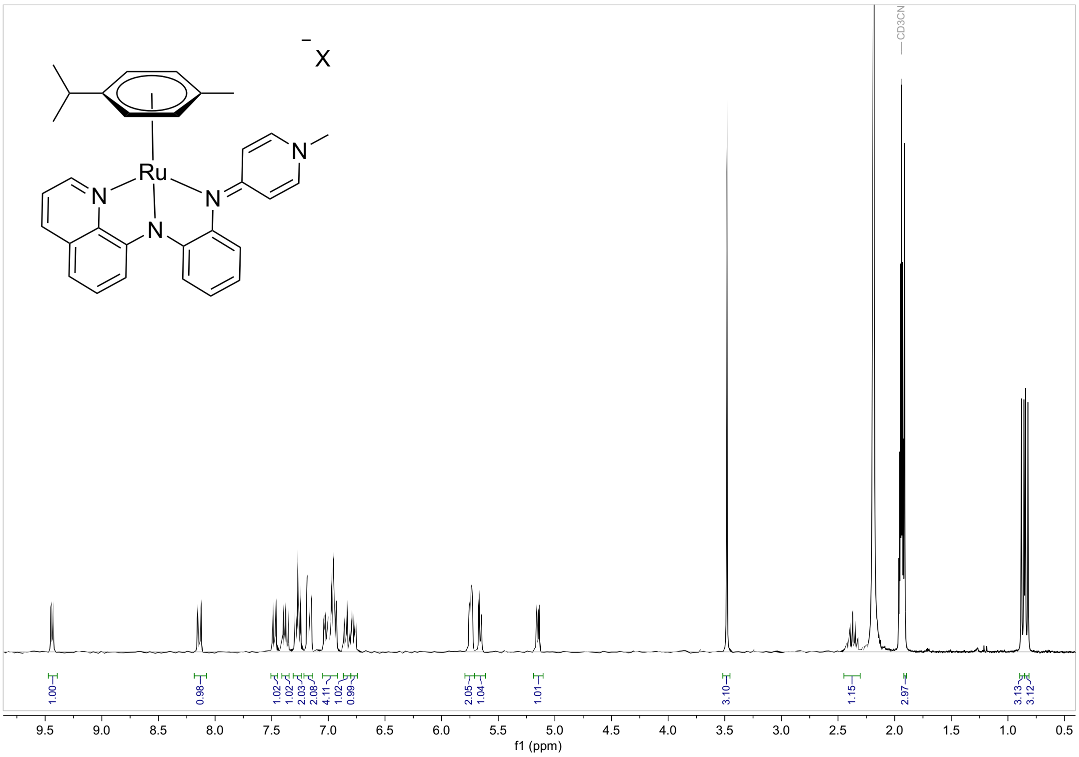

Figure S1. ^{1}H NMR (300MHz, CD_3CN) of **2a**

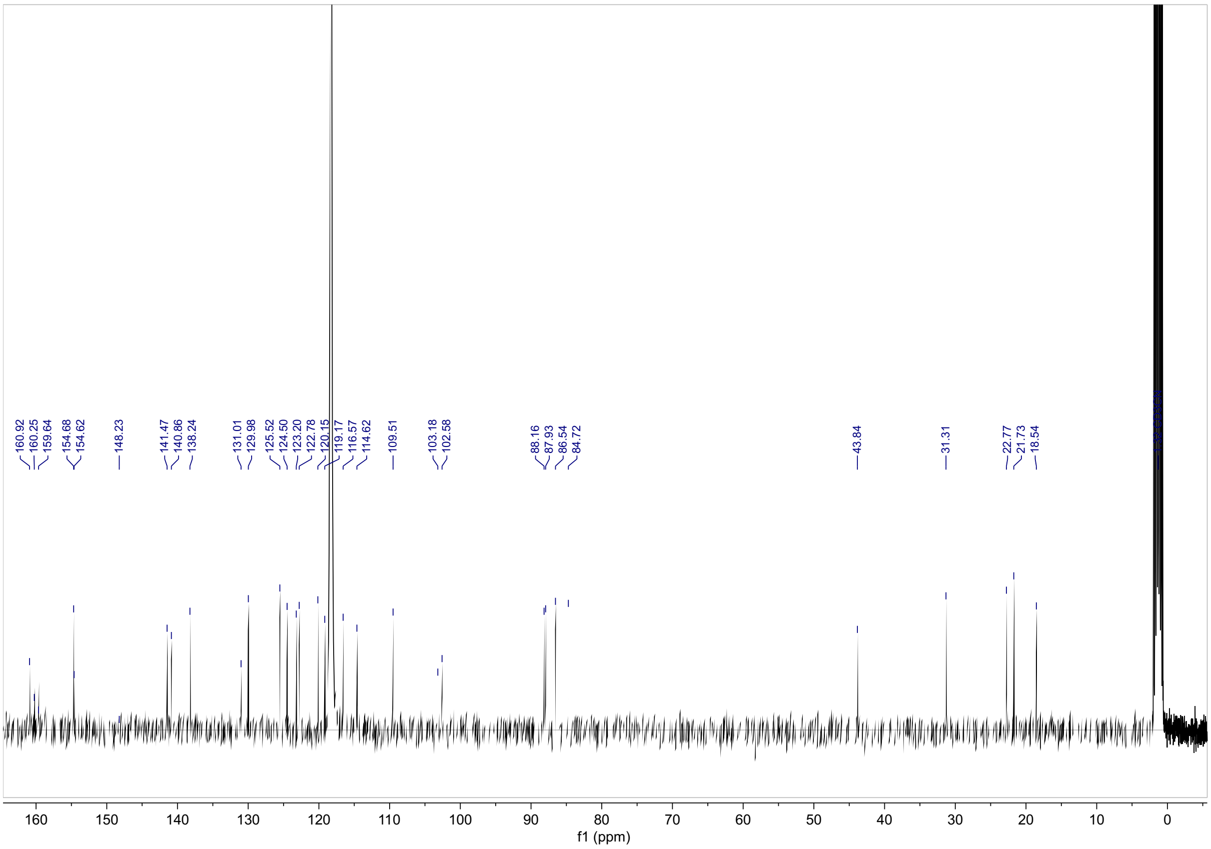

Figure S2. ^{13}C NMR (101MHz, CD_3CN) of **2a**

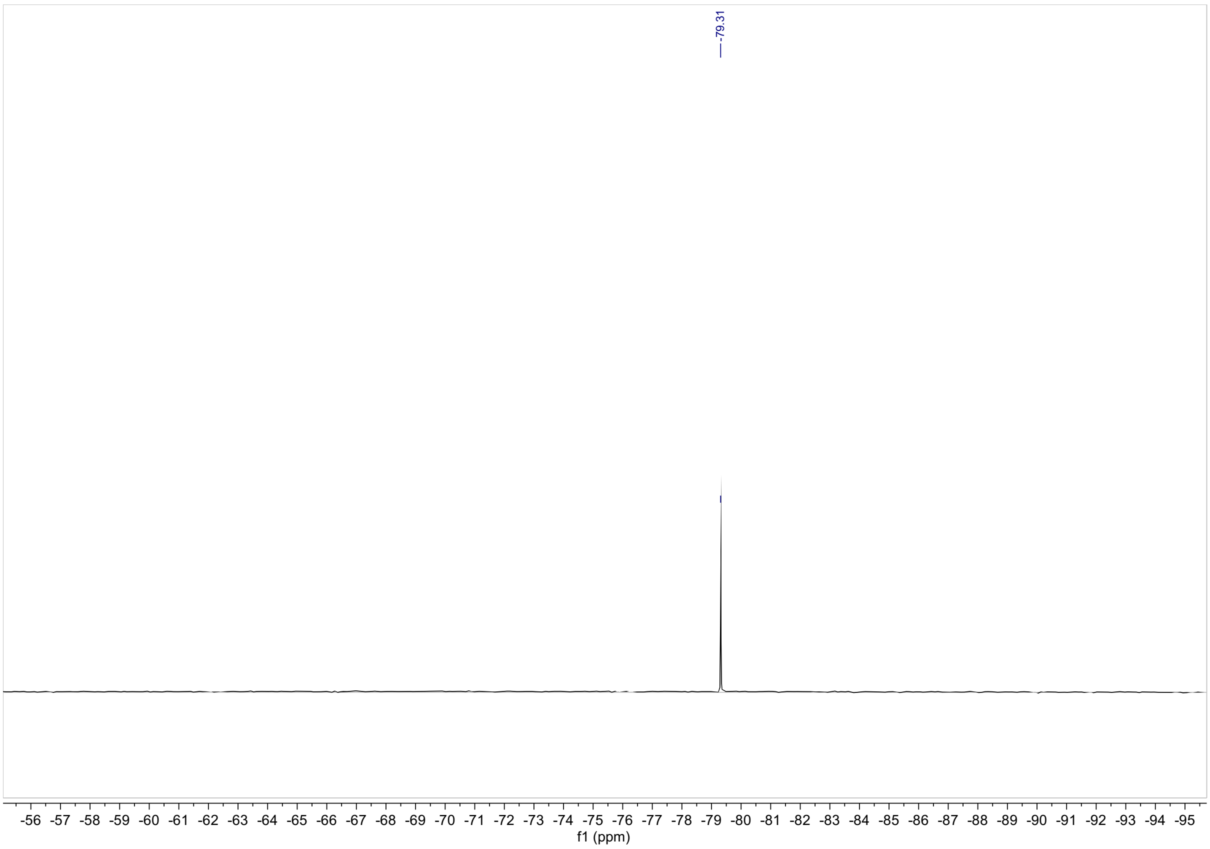

Figure S3. ^{19}F NMR (376 MHz, CD_3CN) of **2a**

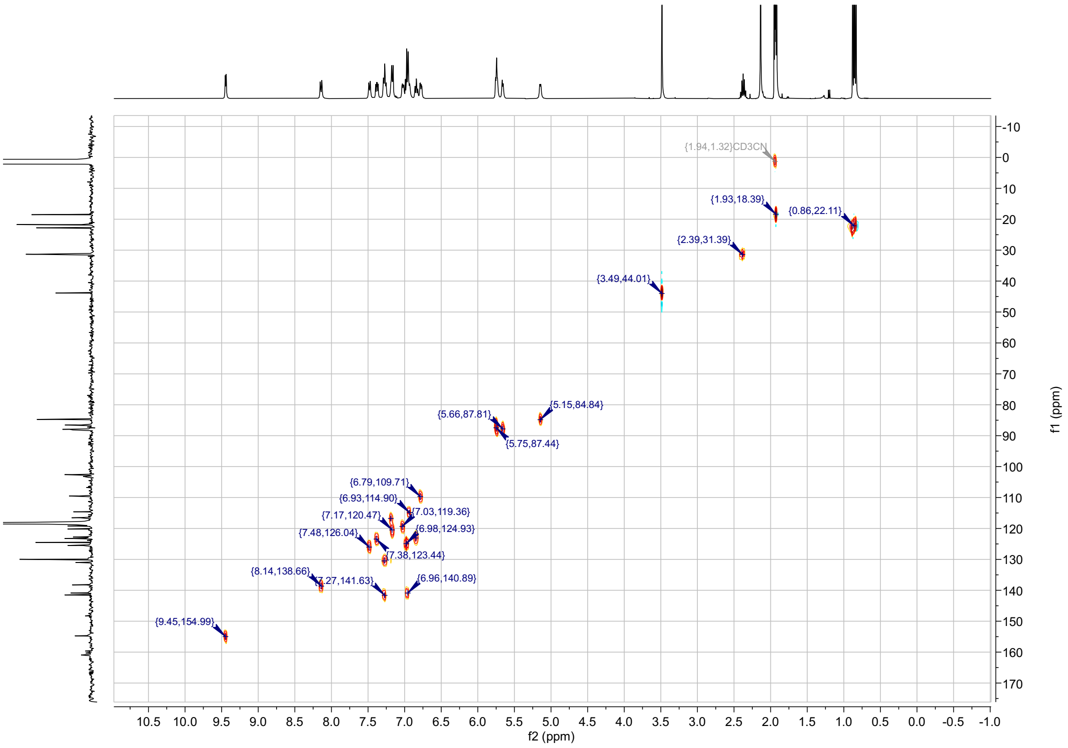

Figure S4. ^{1}H–^{13}C HSQC of **2a**

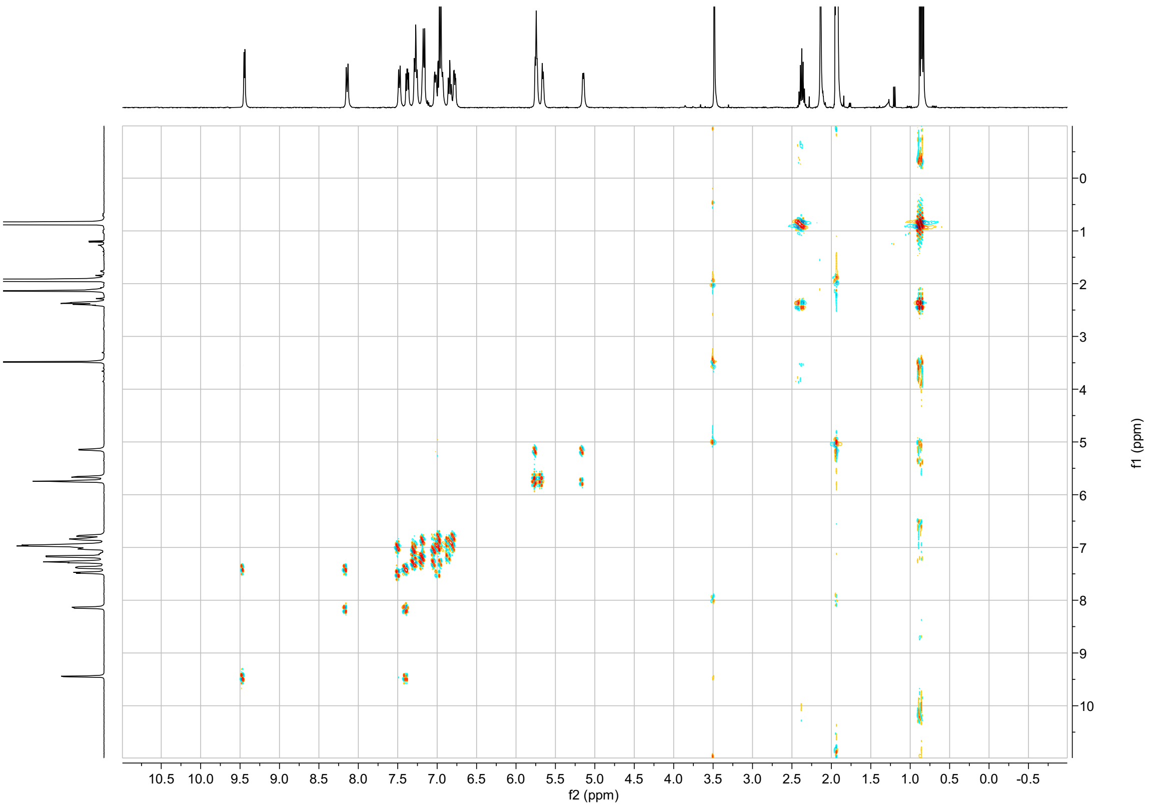

Figure S5. ^{1}H–^{1}H COSY of **2a**

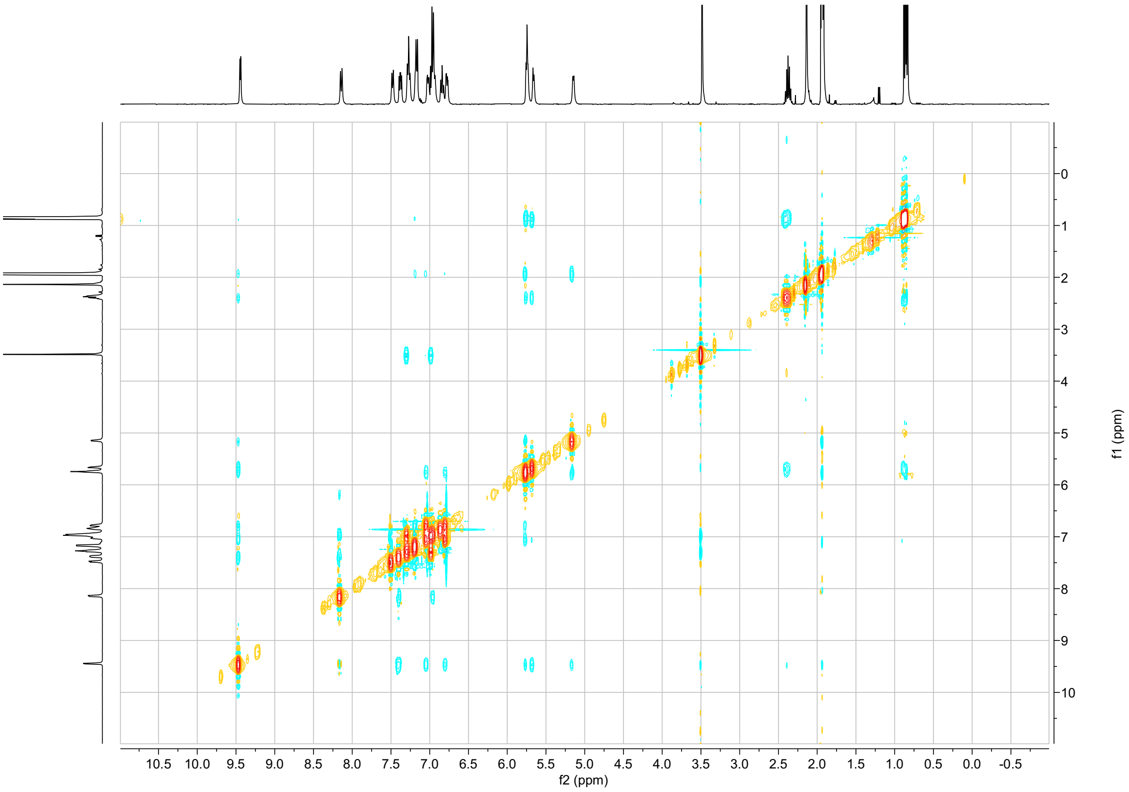

Figure S6. ^{1}H–^{1}H NOESY of **2a**

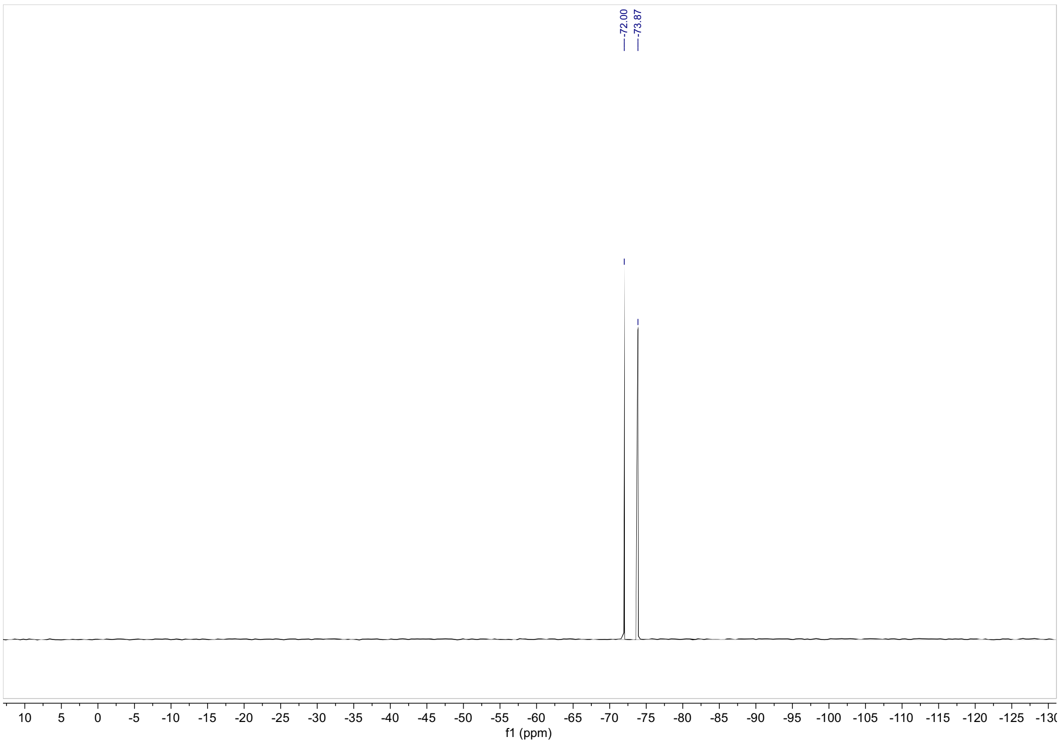

Figure S7. ^{19}F NMR (376 MHz, CD_3CN) of **2b**

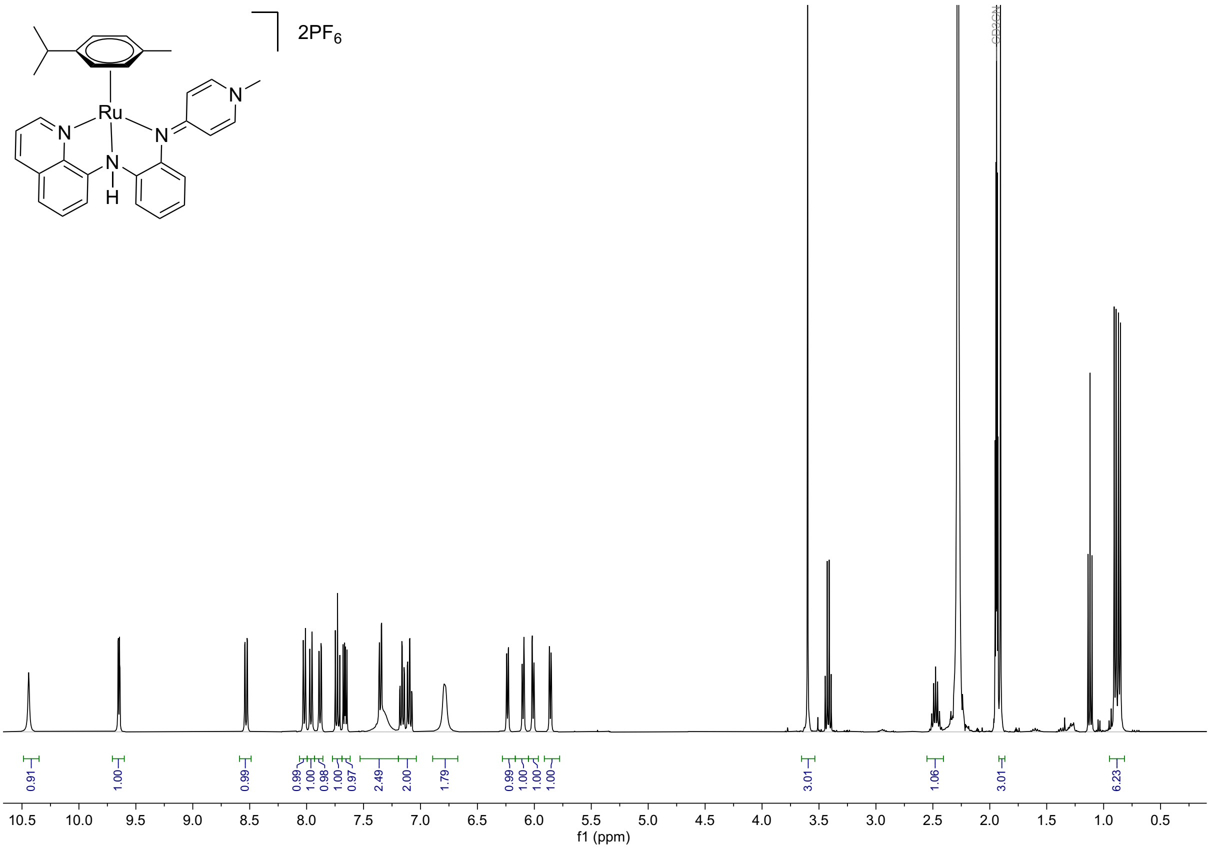

Figure S8. ^{1}H NMR (400MHz, CD_3CN) of **[2a–H]**$^{+}$ with PF_6^{-} counterions.

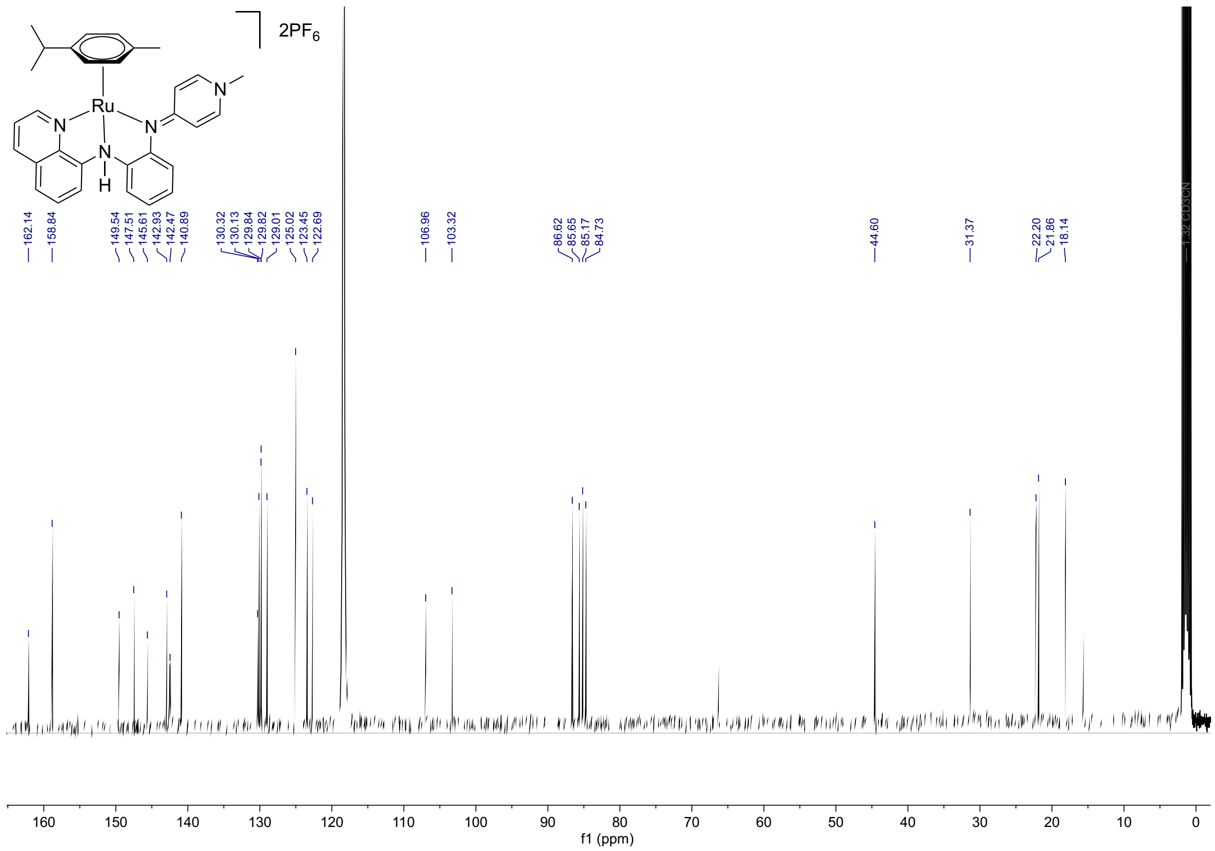

Figure S9. ^{13}C NMR (101MHz, CD_3CN) of **[2a H]**$^+$ with PF_6^- counterions.

3. Reversible Acid-Base reaction

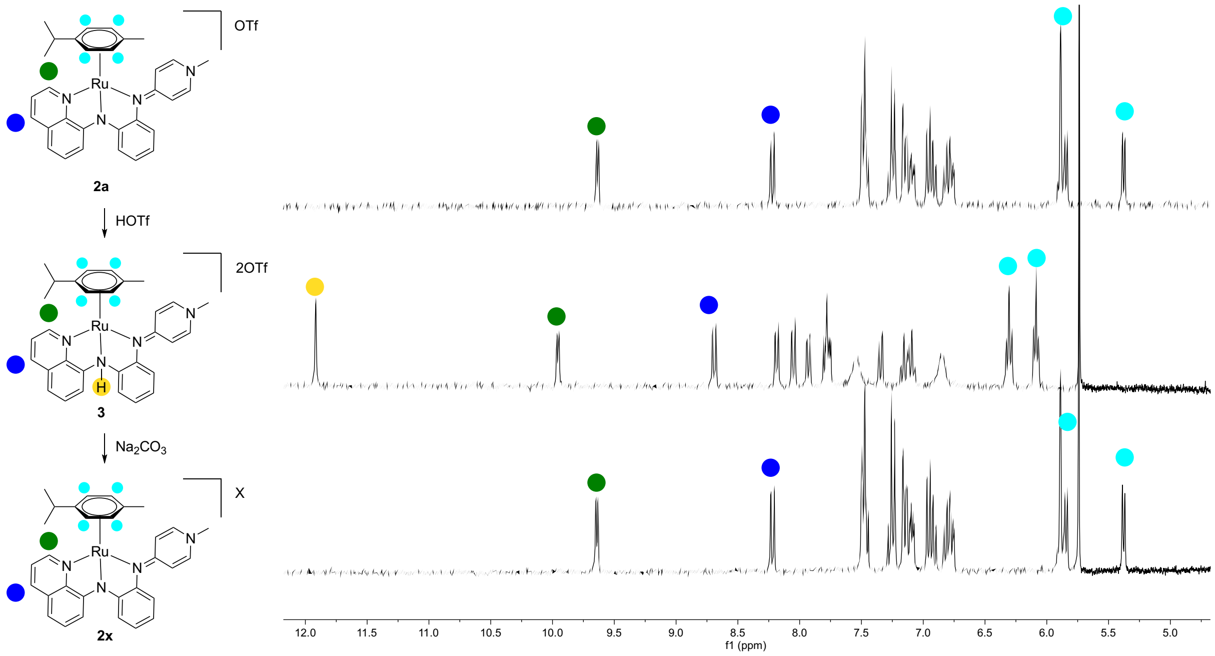

Figure S10. ^{1}H NMR spectra of **2a** (top), of **2a** after addition of HOTf to form complex **[2a–H]**$^+$ (middle), and restoring complex **2a** upon adding Na_2CO_3 (bottom).

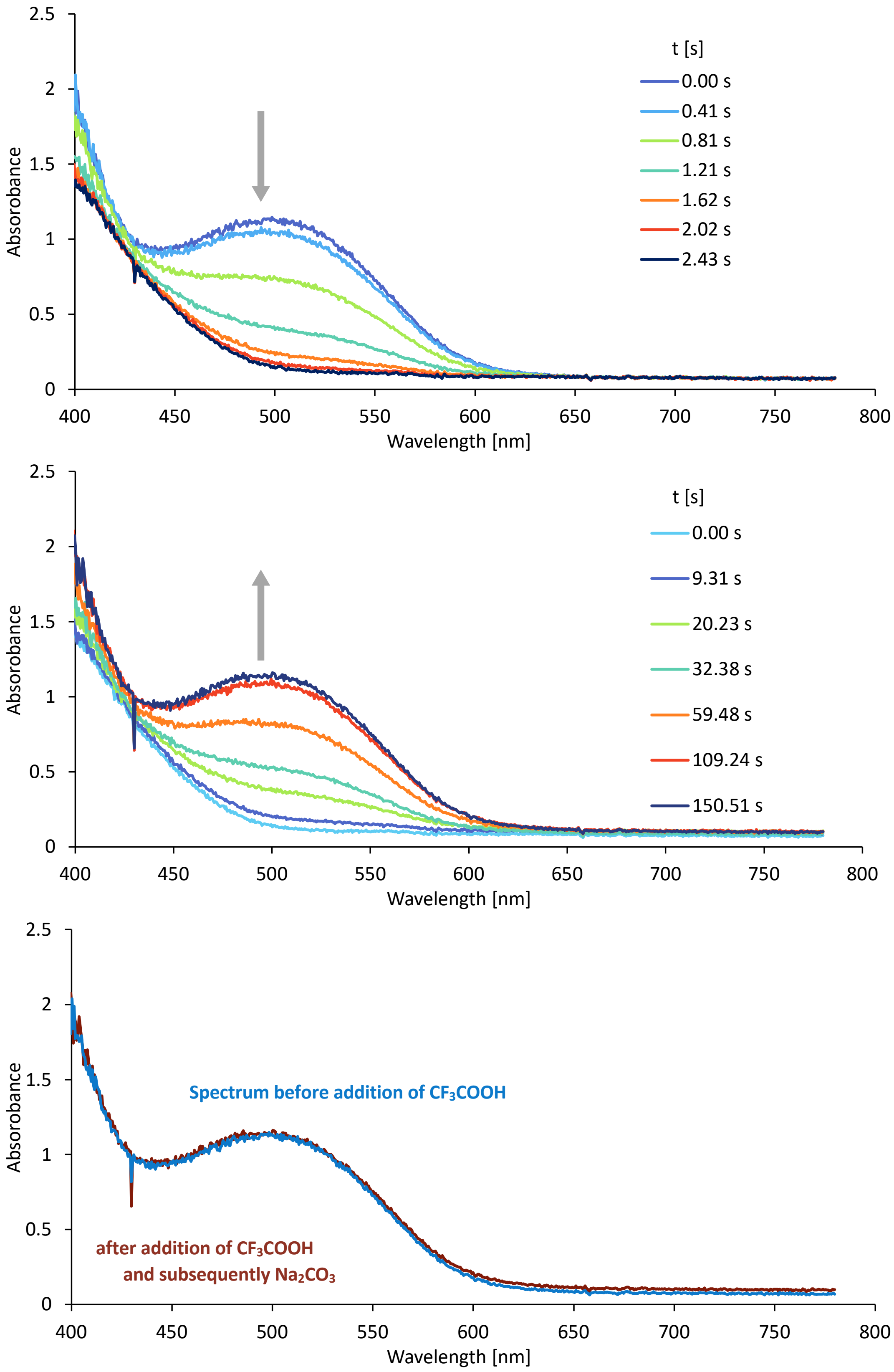

Figure S11. Acid-Base reaction of **2a** monitored by *in situ* UVvis spectroscopy. To a solution of **2a** (7.53mg, 10,6 µmol) in DMSO (10 mL) was added CF_3COOH (50µl, 0.212M in DMSO (*top panel*). Subsequently solid Na_2CO_3 (22.5 mg, 0.2 mmol) was added (*middle panel*). *Bottom panel*: superimposed spectra of complex **2a** before acid addition (blue) and after exposure to CF_3COOH and then Na_2CO_3, revealing spectroscopic identity of the two compounds.

4. Single crystal X-ray diffraction analysis

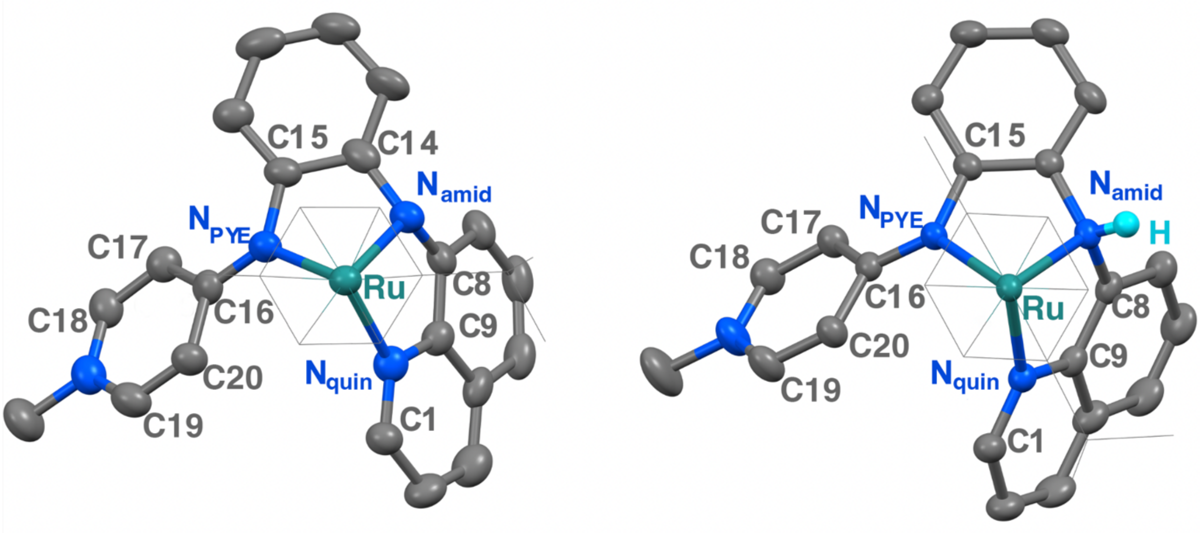

Figure S12. Crystal structure representations of **2a** and **[2a-H]**$^{+}$, perspective down the cymene centroid–Ru axis. All ellipsoids at 50% probability level, PF_6^{-} counteranions and most hydrogen atoms omitted for clarity, p-cym depicted as wireframe for clarity.

Table 1. Selected bond lengths (Å) and angles (deg) for **2a** and **[2a-H]**$^{+}$.

	**2a**	**[2a-H]**$^{+}$
Ru–N$_{quin}$	2.089(6)	2.092(2)
Ru– N$_{amid}$	2.068(5)	2. 132 (3)
Ru–N$_{PYE}$	2.123(6)	2.112(2)
C16–C17	1.41(1)	1.419(3)
C16–C20	1.42(1)	1.420(4)
C17–C18	1.36(1)	1.357(4)
C19–C20	1.35(1)	1.357(4)
N$_{quin}$–Ru–N$_{amid}$	78.6(2)	79.73(8)
N$_{quin}$–Ru–N$_{PYE}$	93.3(2)	85.96(8)
N$_{amid}$–Ru–N$_{PYE}$	75.7(2)	76.66(8)
Ru–N$_{amid}$–C8	114.4(5)	110.8(1)
C8–N$_{amid}$–C10	115.7(6)	112.6(2)
Ru–N$_{amid}$–C10	108.3(5)	107.0(1)

5. Catalytic data

General catalytic procedure

A stock solution of complex **2a** (7.53 mg in 5 mL CH_3CN, 10.6 mM) was prepared under exclusion of light and kept protected from light. An aliquot of the solution (1 mL, 2,13 µmol) was transferred into a 10 mL two-neck round-bottom flask and, under exclusion of light, all volatiles were evaporated under reduced pressure. The flask was equipped with a magnetic stirrer bar and a condenser. The condenser was connected to a three-way valve, connected to a Schlenk line and to a BlueV count volumetric gas measurement device. The round bottom flask and condenser were put under N_2 atmosphere. Under N_2 pressure the valve is opened to the volumetric counter and the tubing flushed with nitrogen. Degassed solvent (1.2 mL) was added to the round bottom flask, the valve to the Schlenk line was closed and the mixture submerged into a preheated oil bath for 10 min. Degassed formic acid (40 µl, 98% purity) was injected (time = 0), and gas evolution was monitored with the BlueV count device.

Influence of anions on catalytic performance

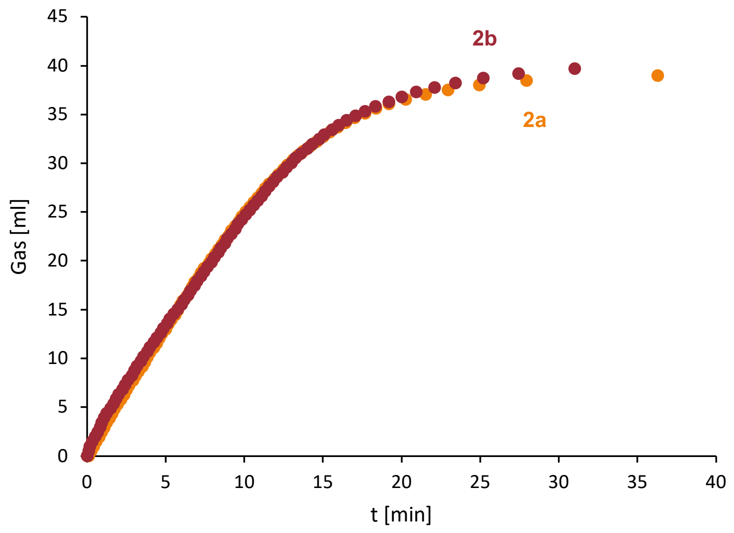

Figure S13. Time-dependent gas evolution profiles for formic acid dehydrogenation with **2a** and **2b.** Reaction conditions: Formic acid (40 µL), [Ru] (0.2 mol%), DMSO (1.2 mL), 80 °C under N_2 atmosphere. Representative single runs are shown, catalytic runs were performed at least twice to ensure reproducibility.

Gas analysis

Gas generated by FA dehydrogenation catalysis was analyzed by gas chromatography (GC 8610C device, SRI Instruments, USA; equipped with a packed Hayesep D column). Argon (99.9999 %, Carba Gas, Switzerland) was used as a carrier gas. CO_2 and CO quantification was carried out with a flame ionization detector (FID) assembled with a methanizer (5 ppm detection limits for CO and CO_2). H_2 was quantified by a thermal conductivity detector (TCD).

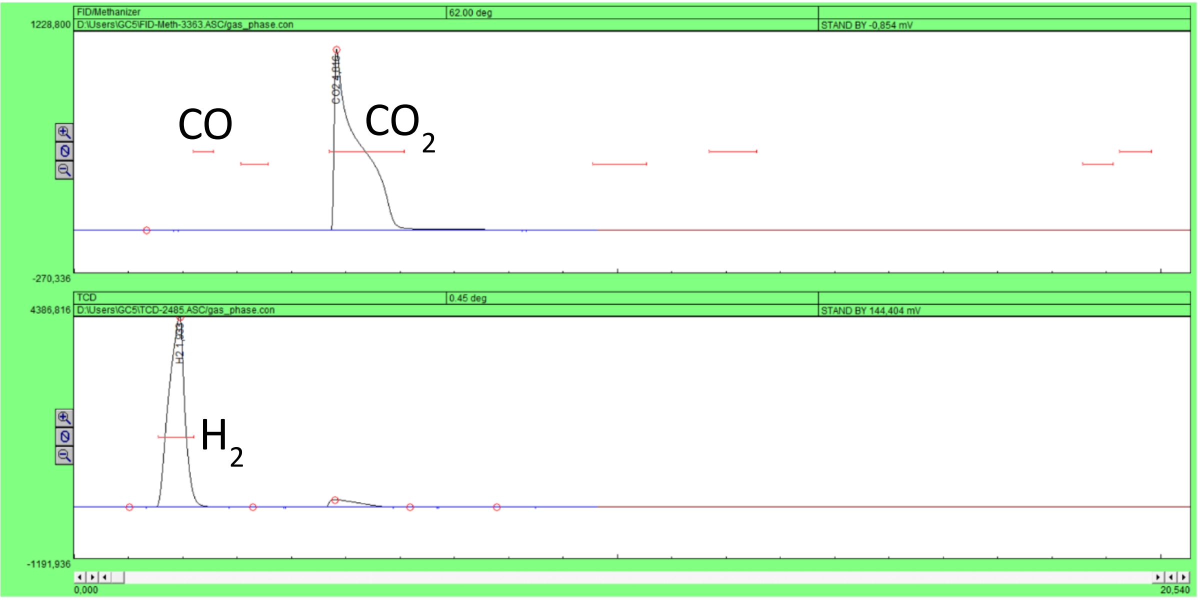

Figure S14: GC trace of the gas mixture of a representative catalytic run. Reaction conditions: **2a** (0.1 mol%, 7.53 mg, 10.6 µmol), FA (200 µl, 5.3 mmol) and DMSO (6 mL) at 30°C. Only CO_2 and H_2 were detected no CO was observed.

FA dehydrogenation catalysis in different solvents

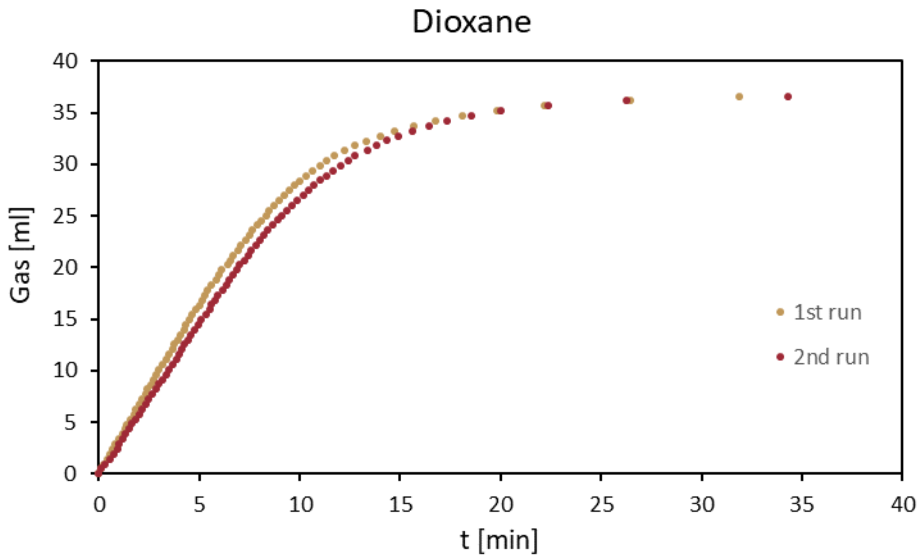

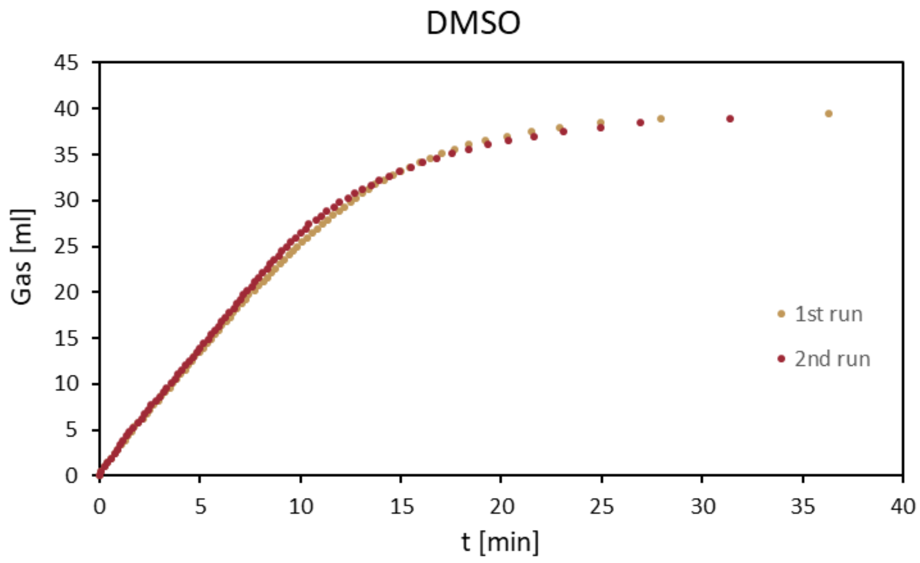

Figure S15. Time-dependent gas evolution profiles for formic acid dehydrogenation with **2a**. Duplicate runs are shown. Reaction conditions: Formic acid (40 µL), [Ru] (0.2 mol%), solvent (1.2 mL), 80 °C under N_2 atmosphere; top: solvent = dioxane, bottom: solvent = DMSO.

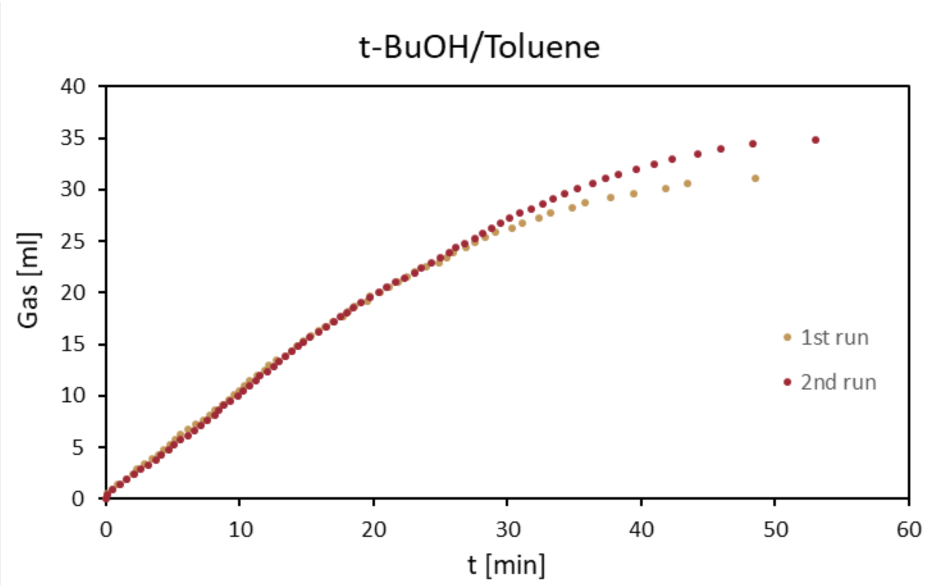

Figure S16. Time-dependent gas evolution profiles for formic acid dehydrogenation with **2a**. Duplicate runs are shown. Reaction conditions: Formic acid (40 µL), [Ru] (0.2 mol%), t-BuOH/Toluene 1:2 (1.2 mL), 80 °C under N_2 atmosphere.

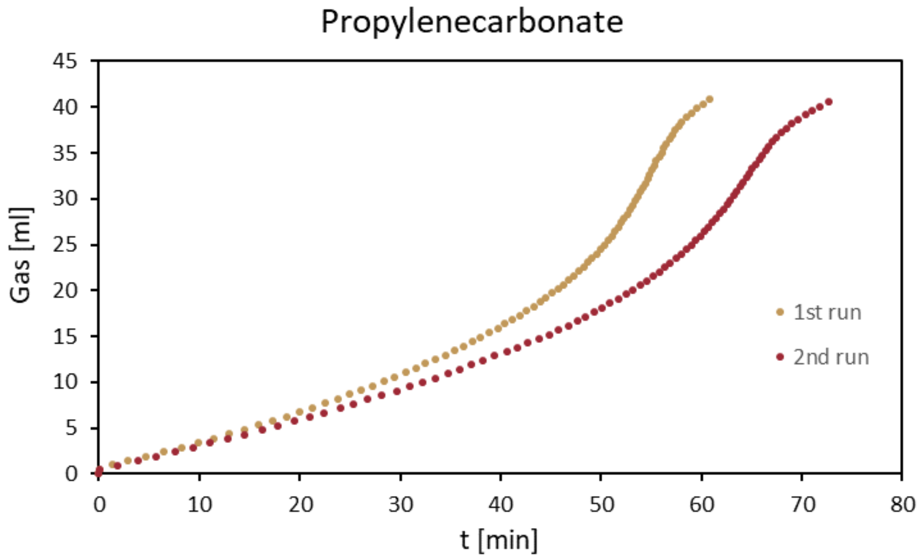

Figure S17. Time-dependent gas evolution profiles for formic acid dehydrogenation with **2a**. Duplicate runs are shown. Reaction conditions: Formic acid (40 µL), [Ru] (0.2 mol%), propylene carbonate (1.2 mL), 80 °C under N_2 atmosphere.

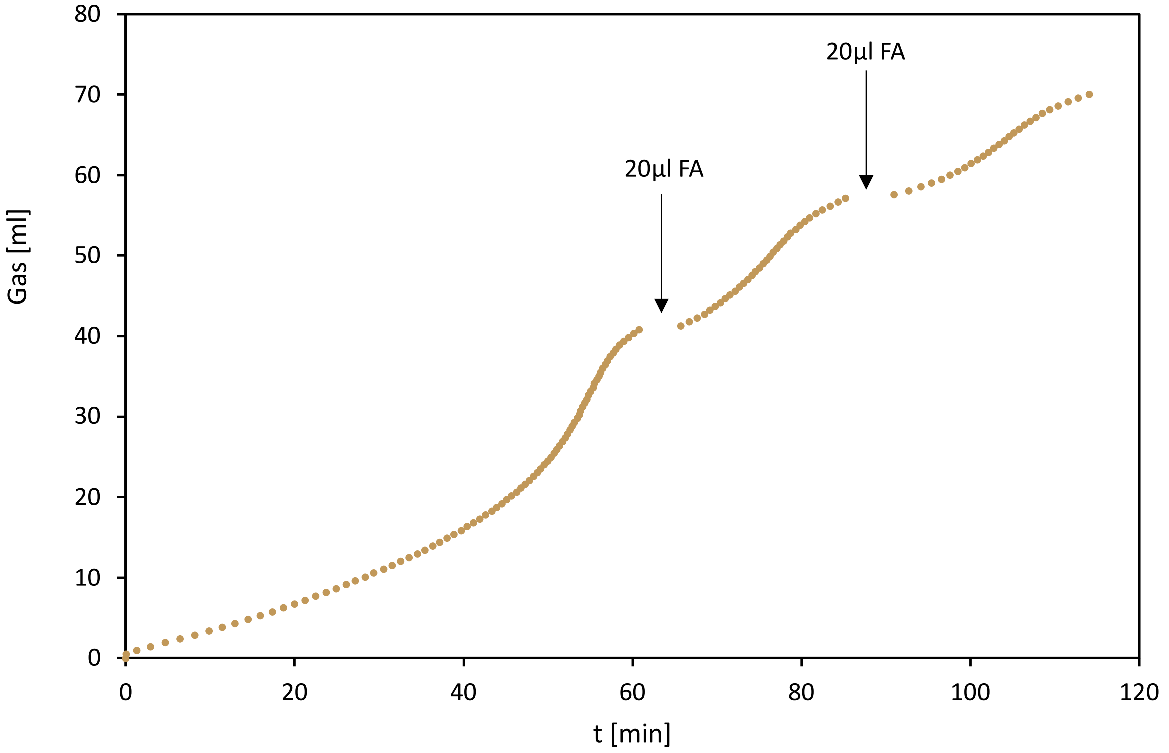

Figure S18. Time-dependent gas evolution profiles in propylene carbonate with repetitive formic acid addition. Reaction conditions: Formic acid (40 µL), **2a** (0.2 mol%), propylene carbonate (1.2 mL), 80 °C under N_2 atmosphere, formic acid additions (20 µL) after 60 and after 90 min.

6. Mechanistic Data

Arrhenius and Eyring analysis

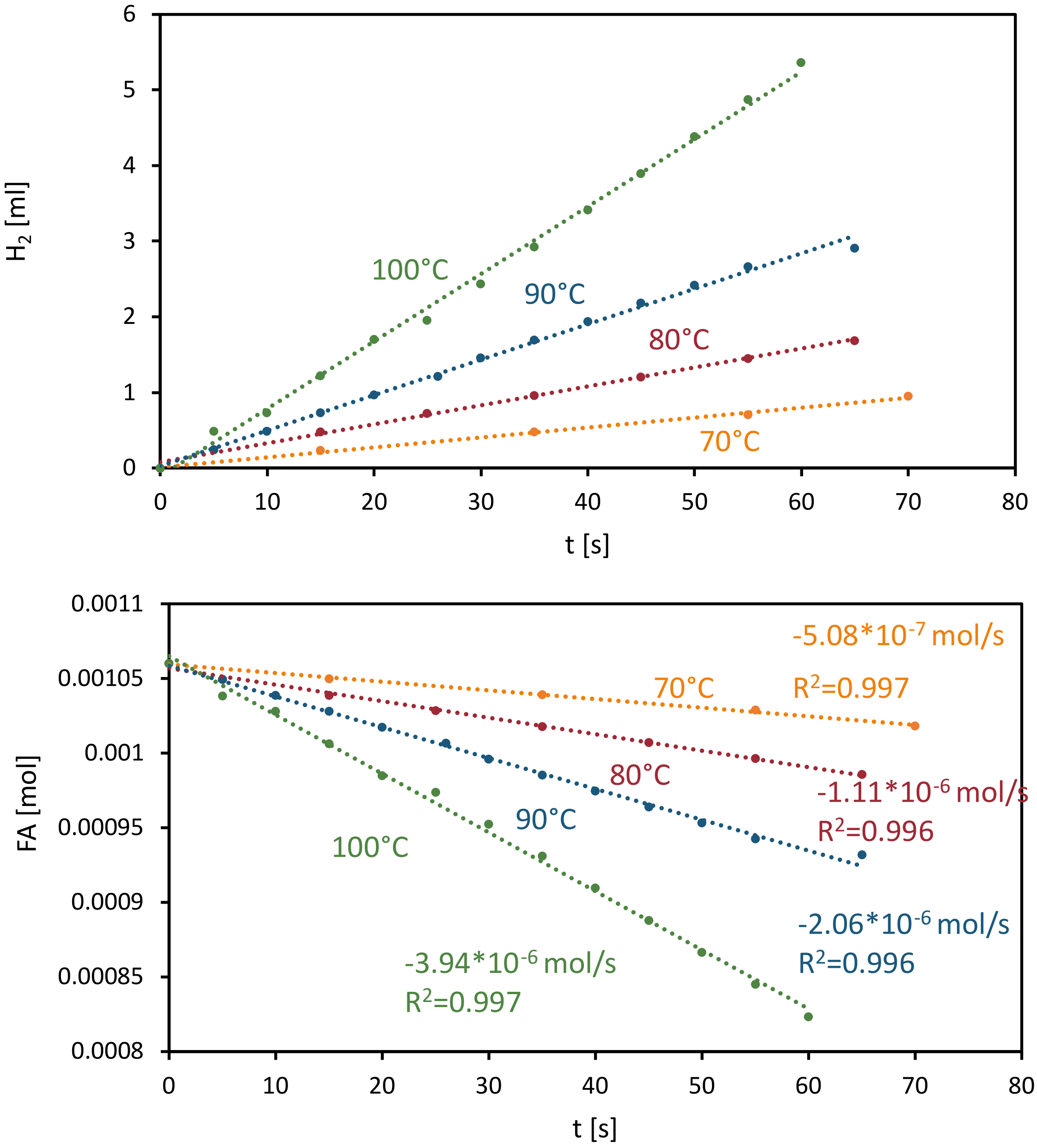

Figure S19: *Top*: Initial production of H_2 at different temperatures. H_2 production was measured with the BlueV count volumetric gas counter. The volume was halved as the evolved gas consists of 1:1 CO_2/H_2 mixture; *bottom*: FA consumption at different temperatures. The FA concentrations were calculated based on initially injected amount minus consumed amount based on evolved gas volume. Reaction condition: **2a** (0.01 mol%), FA (1.06 mmol) and DMSO (1.2 mL) at indicated temperature.

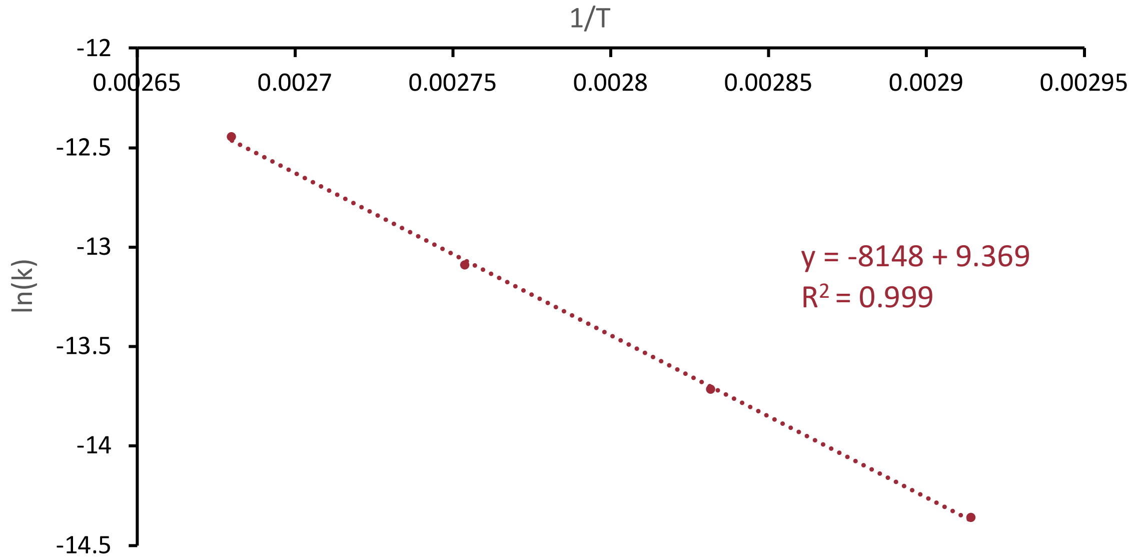

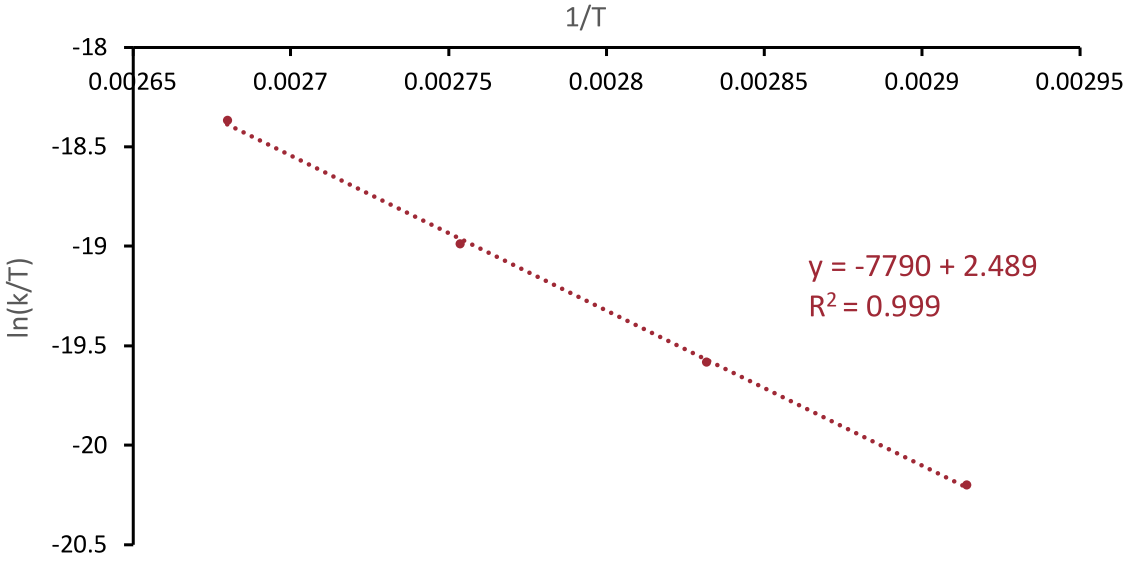

Figure S20. Arrhenius plot (top) and Eyring plot (bottom), form rates deduced from temperature dependent catalysis (see slopes in figure **S19**)

The activation energy E_a was calculated from the slope of the Arrhenius plot:

$$\boldsymbol{E_a} = -\mathrm{R} \times (\text{slope}) = -8.31 \times (-8148) = \mathbf{68\ kJ\ mol^{-1}}$$

The activation parameters $\Delta H^{\ddagger}$ and $\Delta S^{\ddagger}$ were calculated from the slope and intercept of the Eyring plot (with intercept = $\ln(k_B/h) + \Delta S^{\ddagger}/R$):

$$\boldsymbol{\Delta H^{\ddagger}} = -\mathrm{R} \times (\text{slope}) = -8.31 \times (-7790) = \mathbf{65\ kJ\ mol^{-1}}$$

$$\boldsymbol{\Delta S^{\ddagger}} = [(\text{intercept}) - \ln(k_B/h)] \times \mathrm{R} = [2.489 - 23.78] \times 8.31 = \mathbf{-177\ J\ mol^{-1}}$$

In Situ UV vis experiments

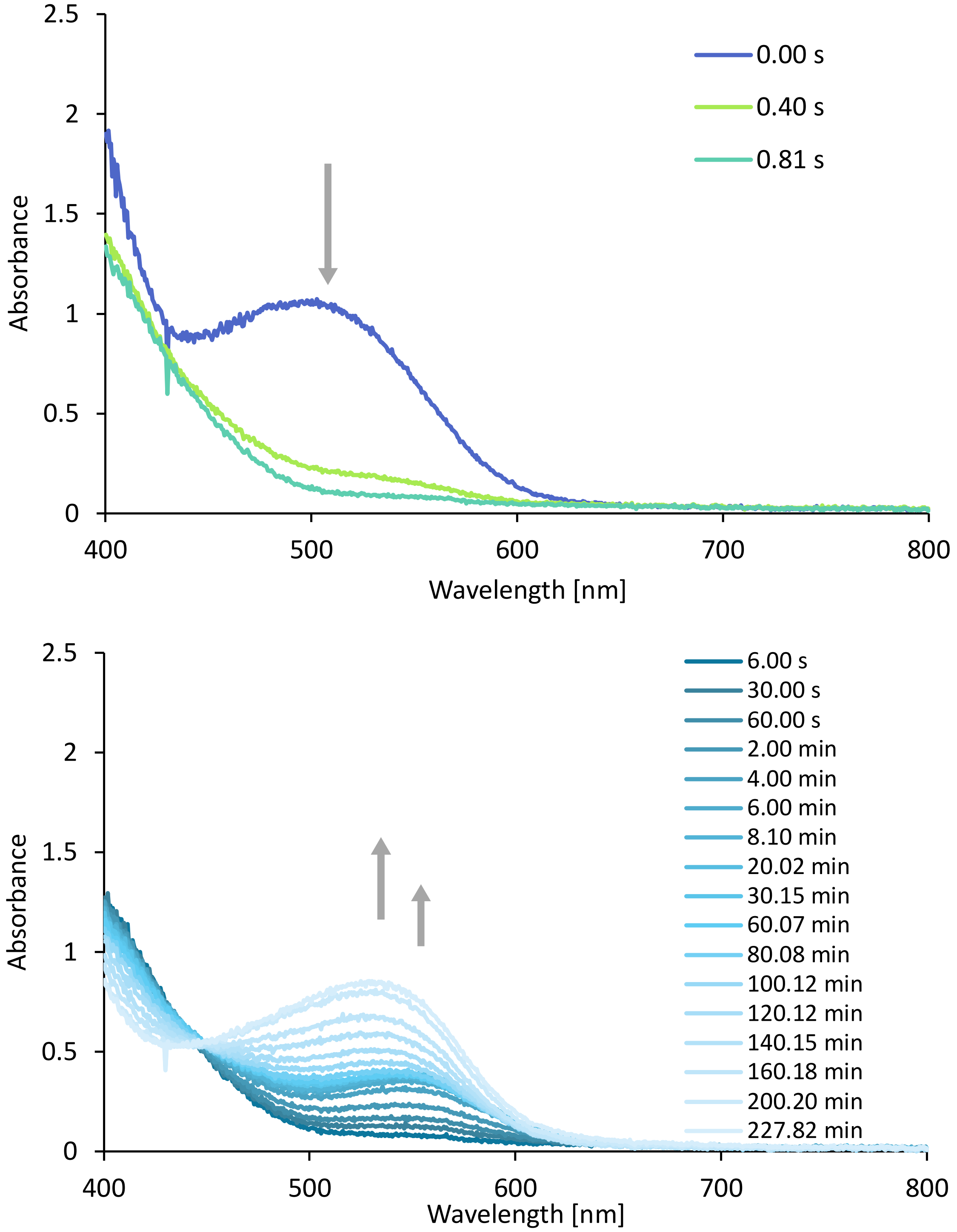

Figure S21. Catalytic reaction at 30 °C monitored by *in situ* UV-vis spectroscopy. Reaction condition: **2a** (0.1 mol%, 7.53 mg, 10.6 µmol), FA (40 µl, 10.6 mmol) and DMSO (10 mL) at 30 °C. Gas evolution was measured concomitantly and is shown in Fig S22.

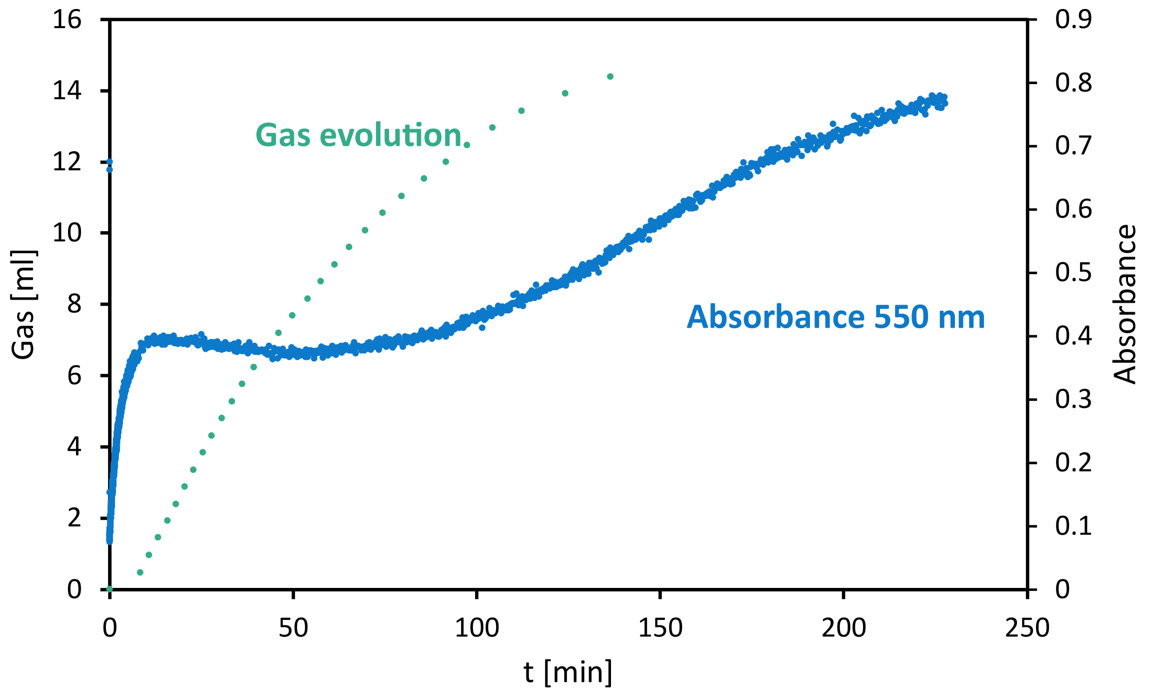

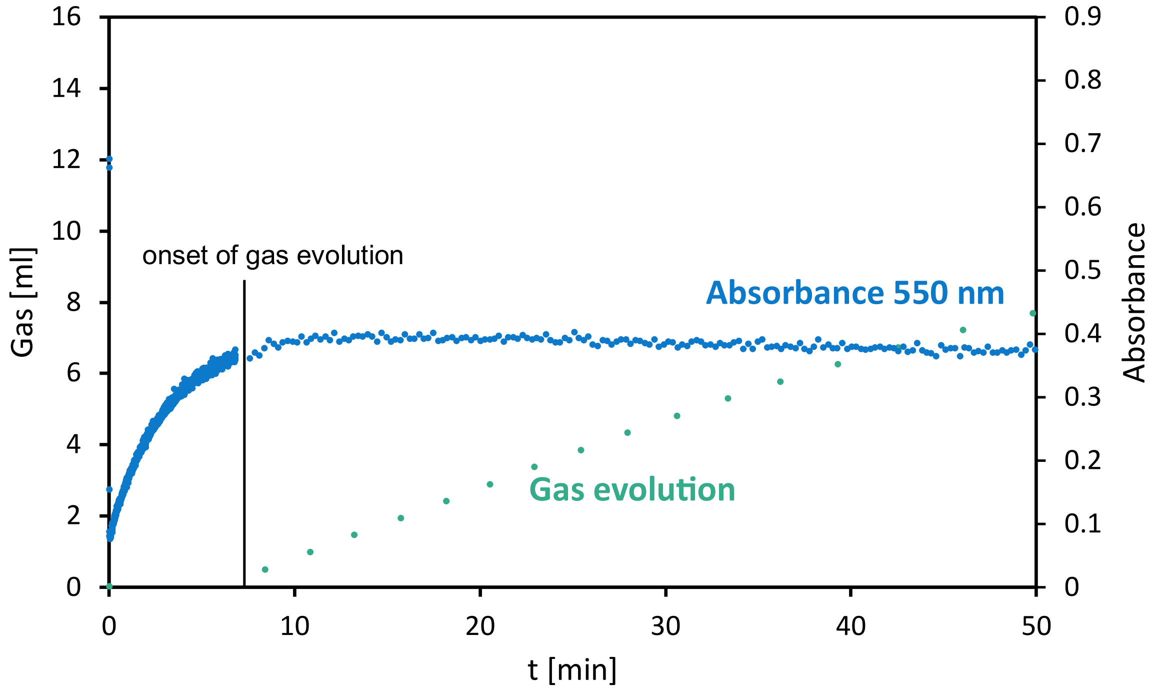

Figure S22: Gas evolution profile *vs* changes in absorption. Full profile (top), expansion of the profile during the inital 50 min (bottom; absorptions at 550 nm extracted from spectra, see Fig. S21).

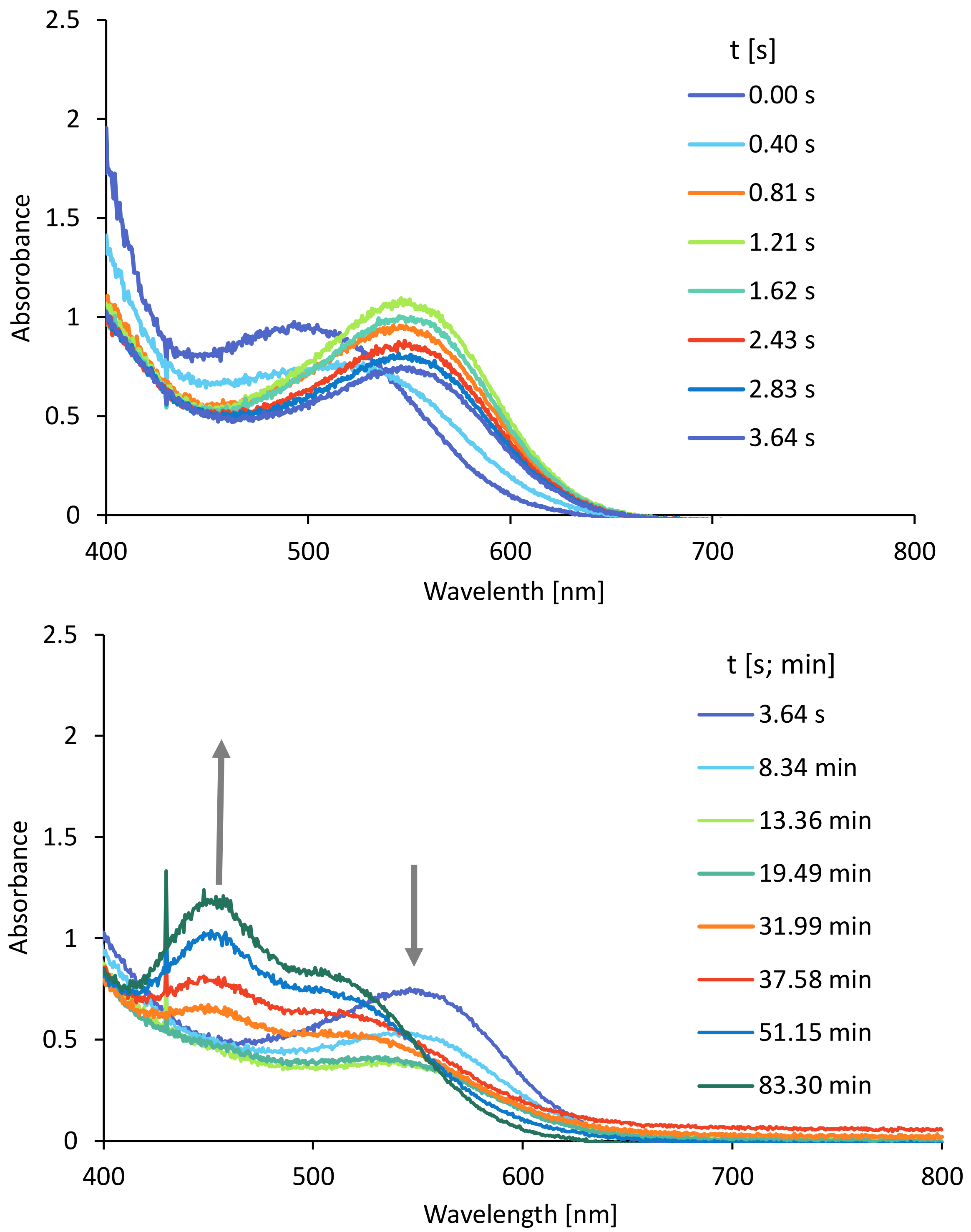

Figure S23. Catalytic reaction at 80 °C followed by in situ UV-vis spectroscopy. Inital reaction (*top panel*), later stages (*bottom panel*). Catalysis was carried out according to general procedure but upscaled to accommodate the UV-probe. Reaction conditions: **2a** (0.1 mol%, 7.53 mg, 10.6 µmol), FA (0.4 mL, 10.6 mmol) and DMSO (10 mL) at 80°C. Gas evolution was measured concomitantly and is shown in Fig. S24**.**

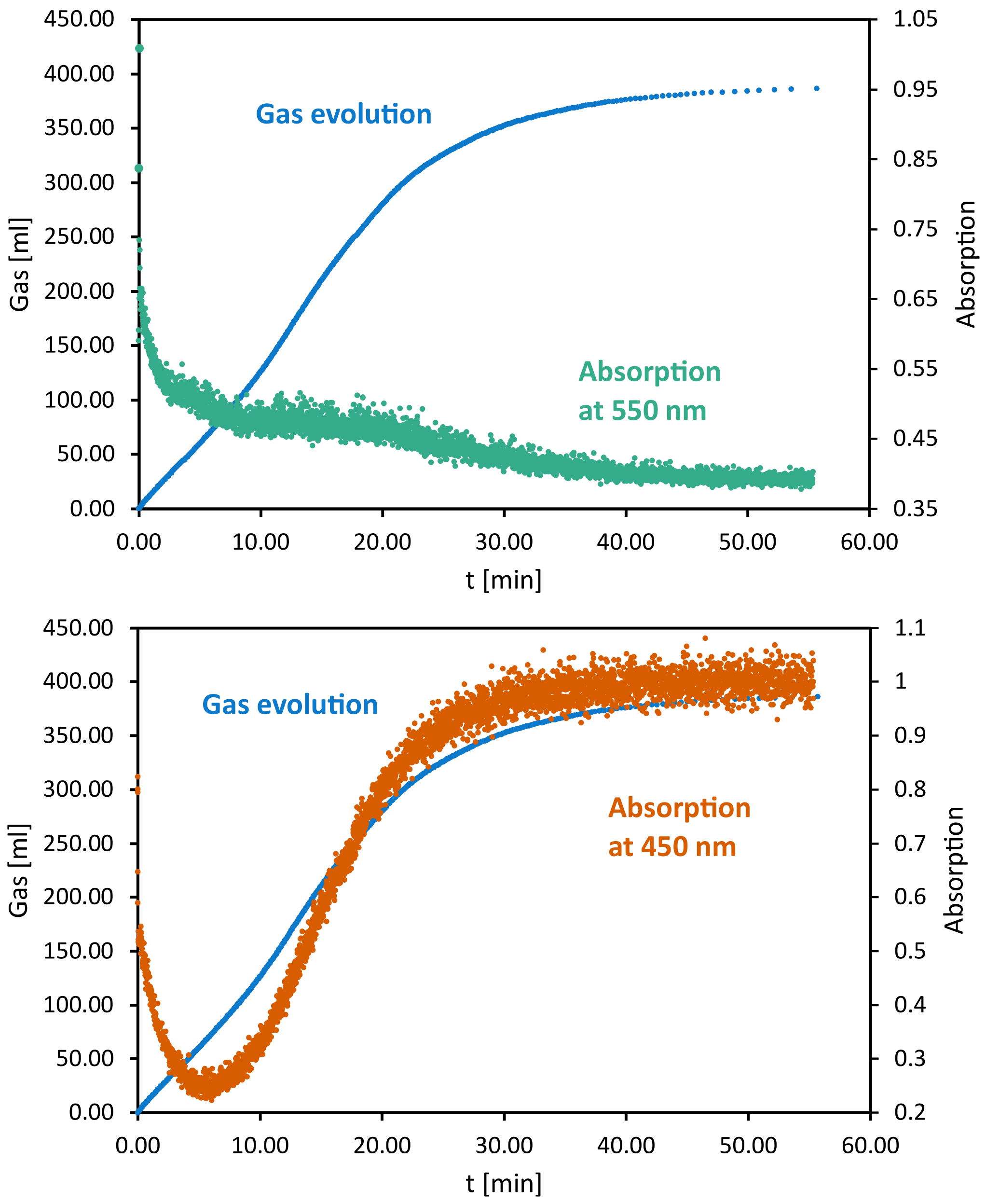

Figure S24. Gas evolution *vs* absorption (absorption data measured in situ, see also Fig. S23).

NMR investigation of FA dehydrogenation reaction

General considerations: The following experiments are prepared under N_2 atmosphere. FA addition was however carried out in close proximity to the NMR spectrometer and the tube was opened to air during the addition.

Stochiometric experiments

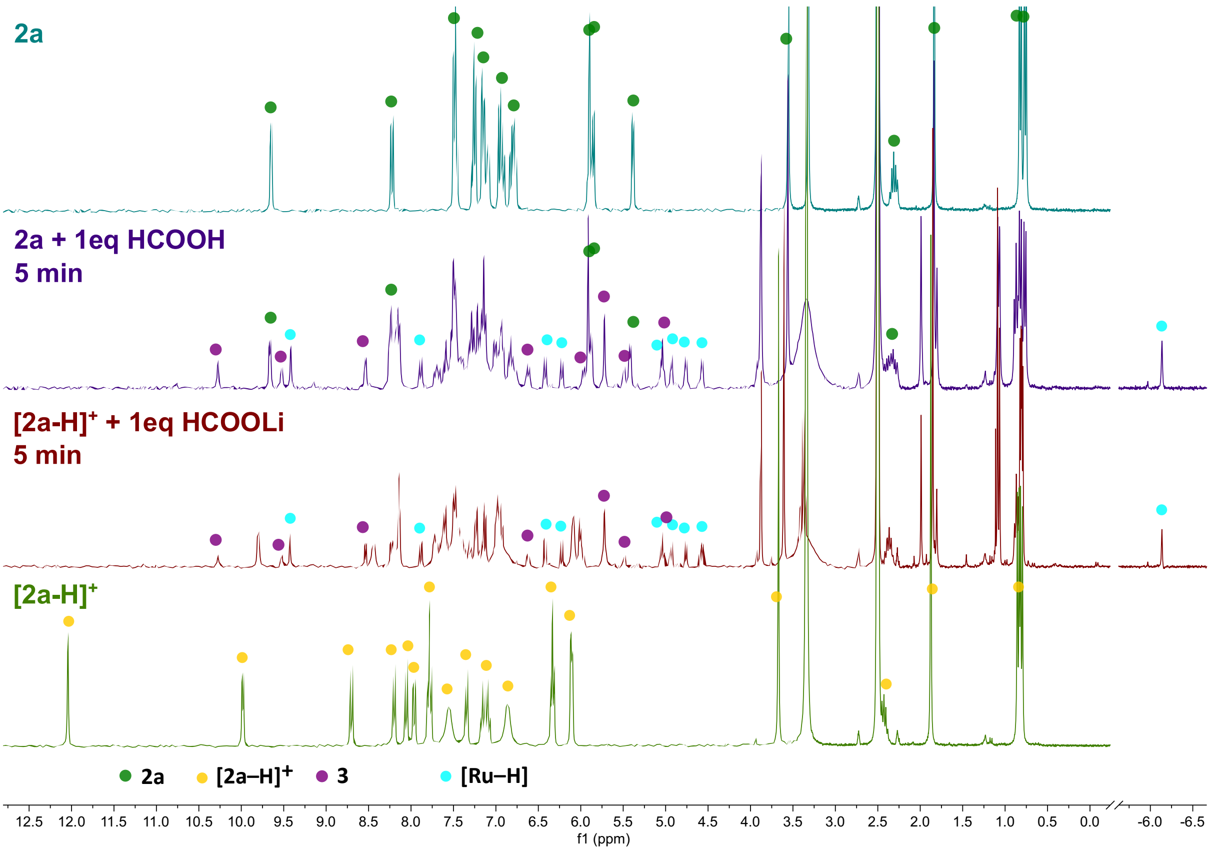

Figure S25. Stacked ^{1}H NMR spectra of stochiometric experiments with FA/ lithium formate. Reaction conditions: *Top*: **2a** (4 mg, 5.64 µmol), DMSO-d_6 (0.6 mL); *2nd spectra*: Addition of 1eq FA to a solution of **2a** (4 mg, 5.64 µmol), DMSO-d_6 (0.6 mL), FA (5.64 µmol in 10 µl DMSO-d_6). *3rd spectra*: To a solution of**[2a-H][2PF₆]** (4.8 mg, 5.64 µmol) in DMSO-d_6 1eq LiHCOO (5.64 µmol in 100 µl DMSO-d_6) was added. Bottom: **[2a-H]**$^+$ in DMSO-d_6. Signals assigned to **3** (•), **[2a-H]**$^+$ (•), **[2a]** (•) and the **[Ru-H]** (•) are marked.

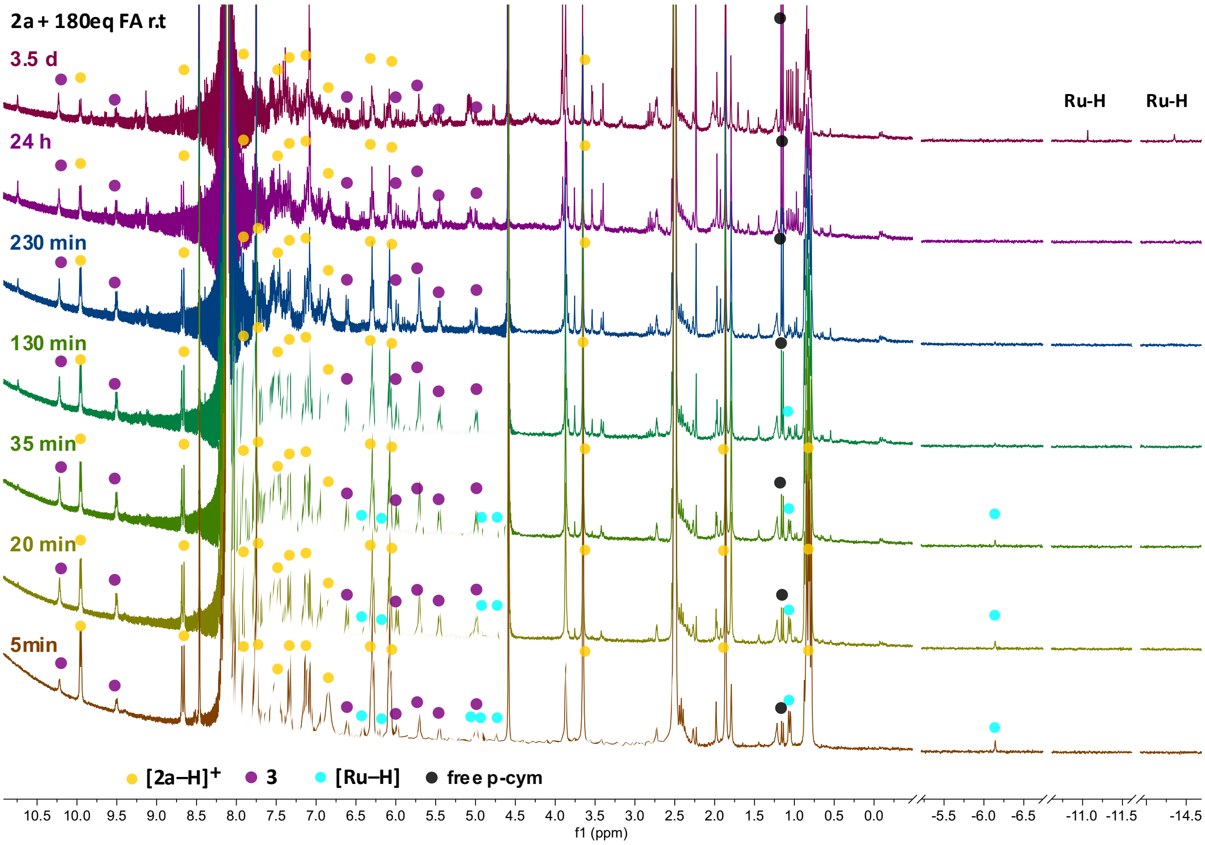

Figure S26. Stacked ^1H NMR spectra after addition of 180 eq FA to **2a**. Conditions: DMSO-d_6 (0.6 mL), **2a** (4 mg, 5.64 µmol), FA (40 µl, 1.06 mmol). Spectrum immediately after addition of FA (t = 4 min) shown in Fig. S27. Signals assigned to **3** (•), **[2a-H]**$^+$ (•) and the **[Ru-H]** (•) are marked.

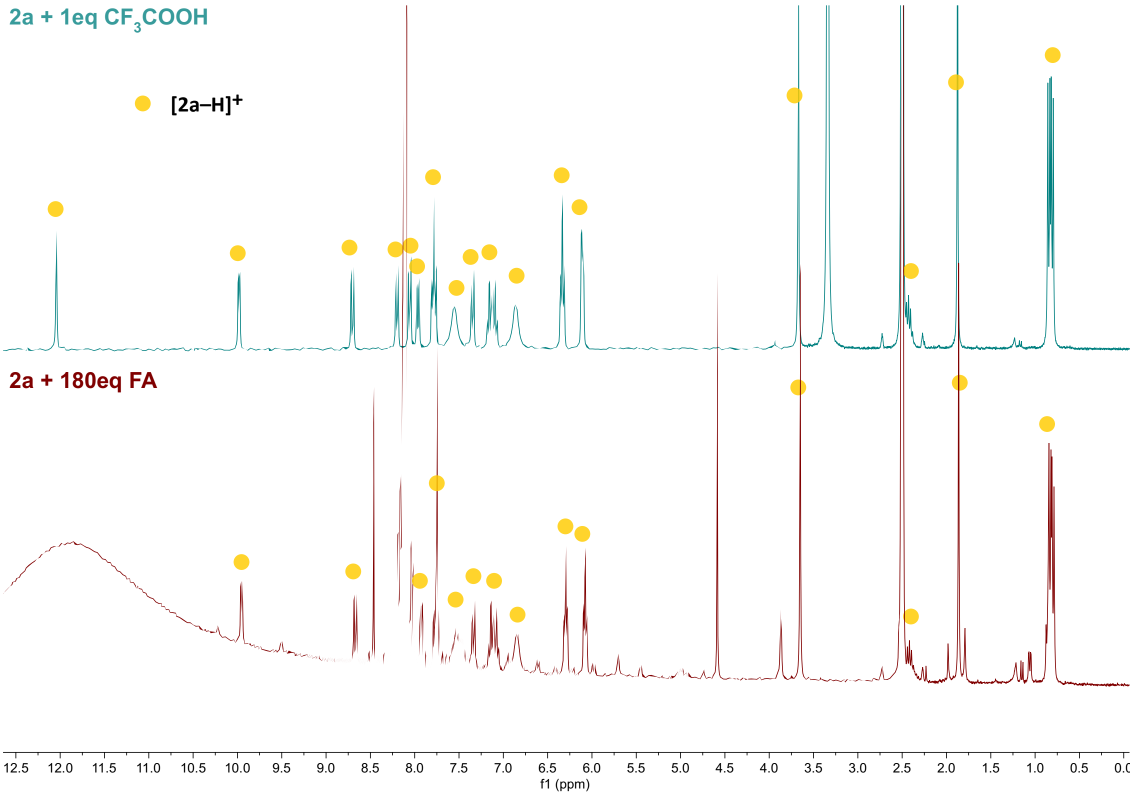

Figure S27. Stacked ^1H NMR spectra of protonated complex **[2a–H]**$^+$ formed after addition of either trifluoroacetic acid (top) or after addition of 180 eq FA (bottom) to complex **2a** (4 min in Figure S26).

Conditions *top*: DMSO-d6 (0.6 mL), **2a** (4 mg, 5.64 µmol), CF_3COOH (5.64 µmol in 10 µl DMSO-d6). Conditions *bottom*: DMSO-d6 (0.6 mL), **2a** (4 mg, 5.64 µmol), FA (40 µl, 1.06 mmol).

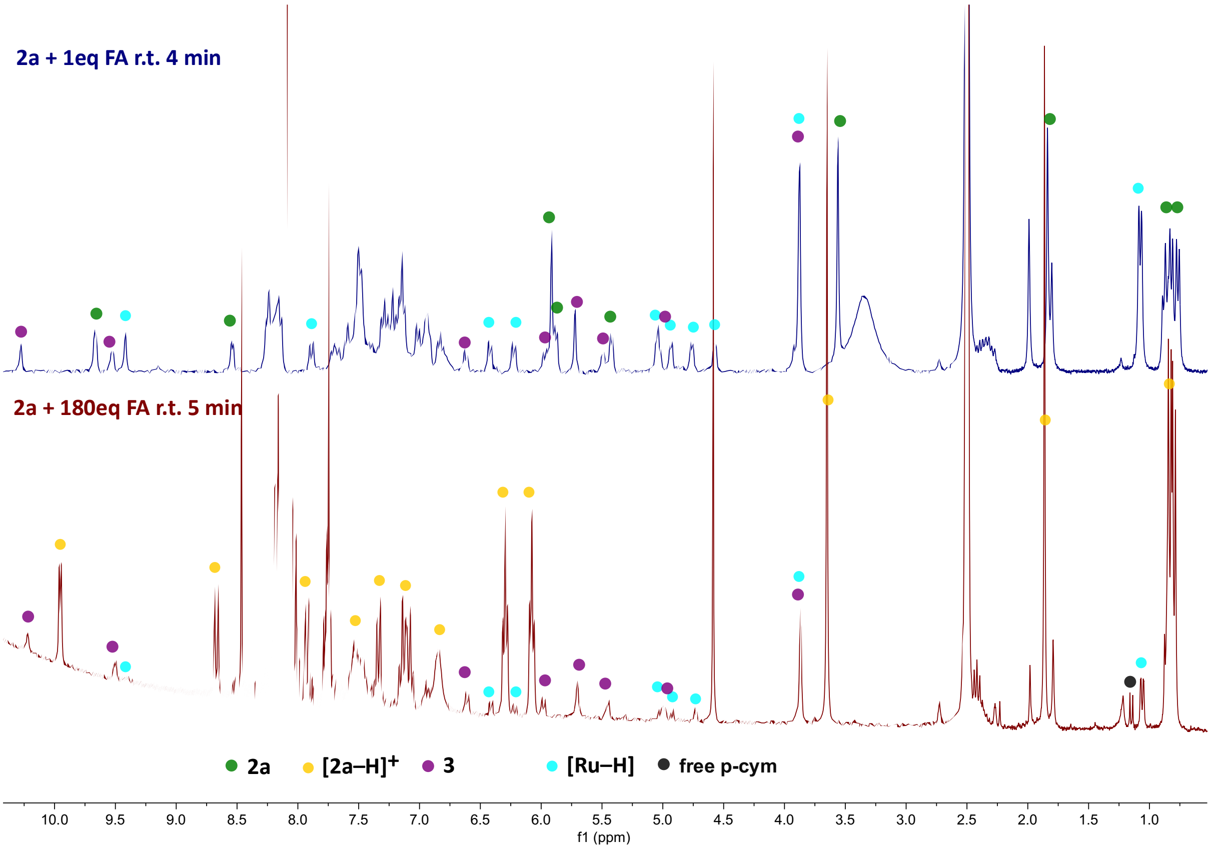

Figure S28. Comparative ^{1}H NMR spectra of **2a** treated with different amounts of FA. *Top*: taken from Fig. S25, DMSO-d6 (0.6 mL), **2a** (4 mg, 5.64 µmol), FA (5.64 µmol in 10 µl DMSO-d6); *bottom*: taken from Fig. S26, DMSO-d6 (0.6 mL), **2a** (4 mg, 5.64 µmol), FA (40 µl, 1.06 mmol). Signals assigned to **3** (•), **[2a-H]**$^+$ (•), **[2a]** (•), free p-cym (•) and the **[Ru-H]** (•) are marked.

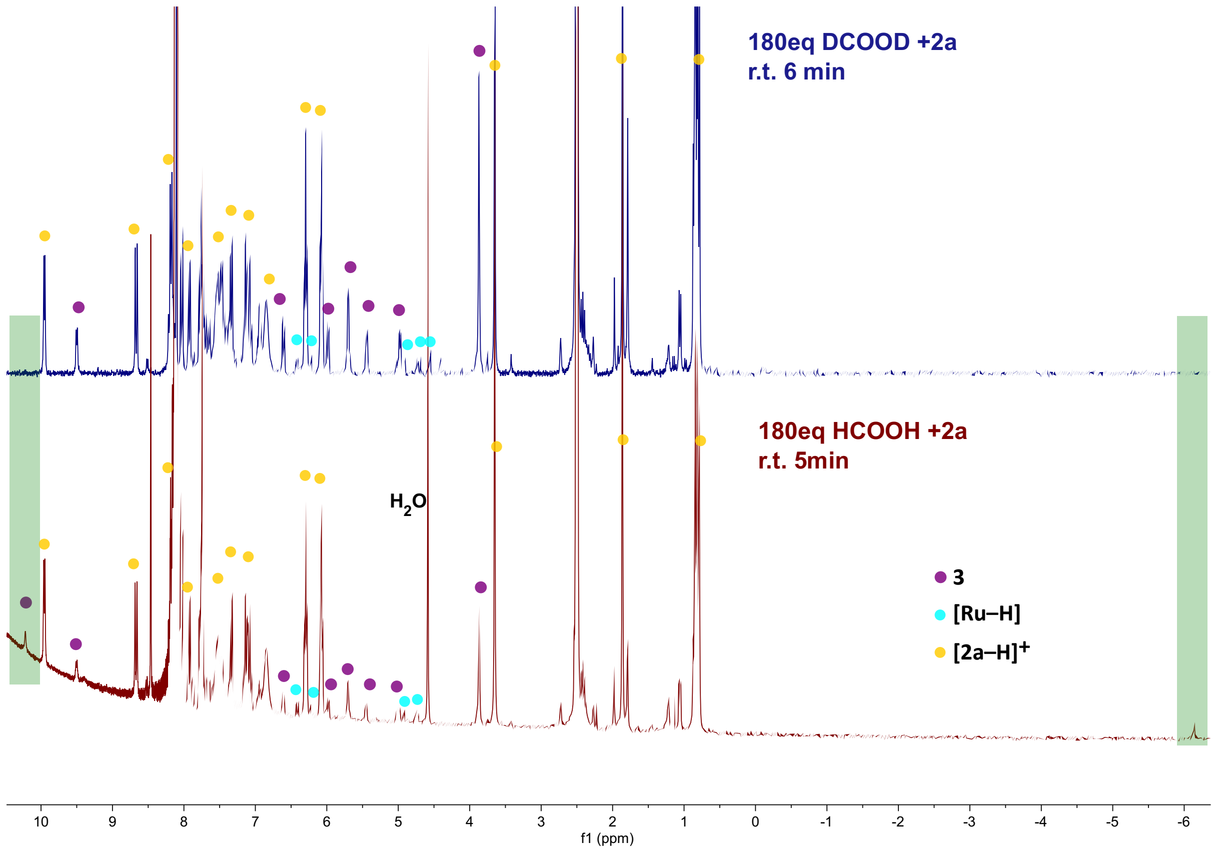

Figure S29. Stacked ^{1}H NMR spectra of **2a** treated with FA or deuterated FA. Top spectrum: DMSO-d_6 (0.6 mL), **2a** (4 mg, 5.64 µmol), DCOOD (40 µl, 1.06 mmol), spectrum shown 6 min after reaction start. Bottom spectrum: DMSO-d_6 (0.6 mL), **2a** (4 mg, 5.64 µmol), FA (40 µl, 1.06 mmol) spectrum shown 4 min after reaction start (see Fig. S26). Signals assigned to **3** (•), **[2a-H]**$^+$ (•), and the **[Ru-H]** (•) are marked. Resonances that are not apparent with DCOOD are highlighted in green.

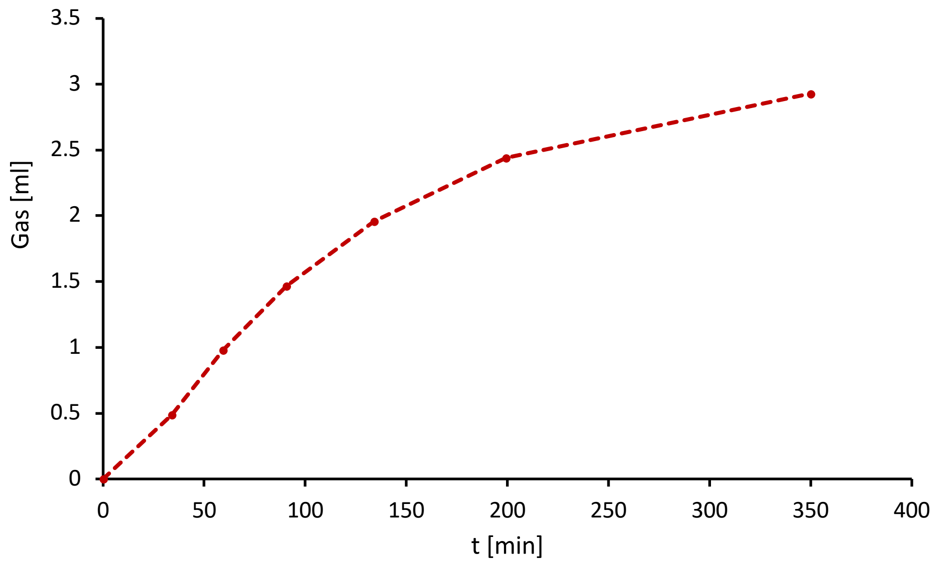

Figure S30. Gas evolution from NMR tube with deuterated FA. Usual preparation of NMR sample but the tube was connected to a blueVcount volumetric gas measurement device. Conditions: DMSO-d_6 (0.6 mL), **2a** (4 mg, 5.64 µmol), DCOOD (40 µL, 1.06 mmol), r.t.

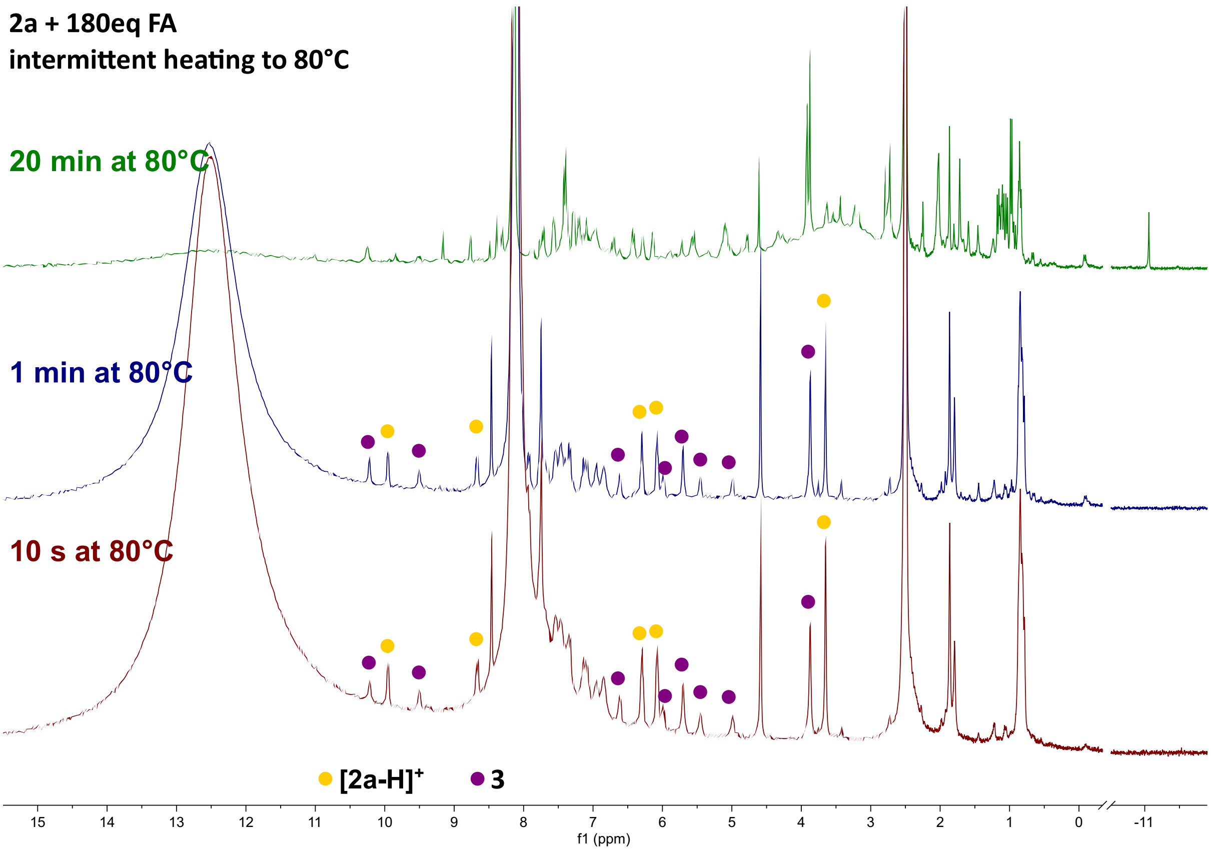

Figure S31. Stacked ^{1}H NMR spectra after addition of 180eq FA to **2a**. Conditions: DMSO-d_6 (0.6 mL), **2a** (4 mg, 5.64 µmol), FA (40 µl, 1.06 mmol). Experiment was carried out in a Young NMR tube under N_2 atm. Indicated times correspond to time in oilbath during which the tube was slightly opened to allow gas to escape. For the measurements the tube was closed, cooled to r.t. and immediately measured. Signals assigned to **3** (●) and **[2a-H]**$^+$ (●) are marked.

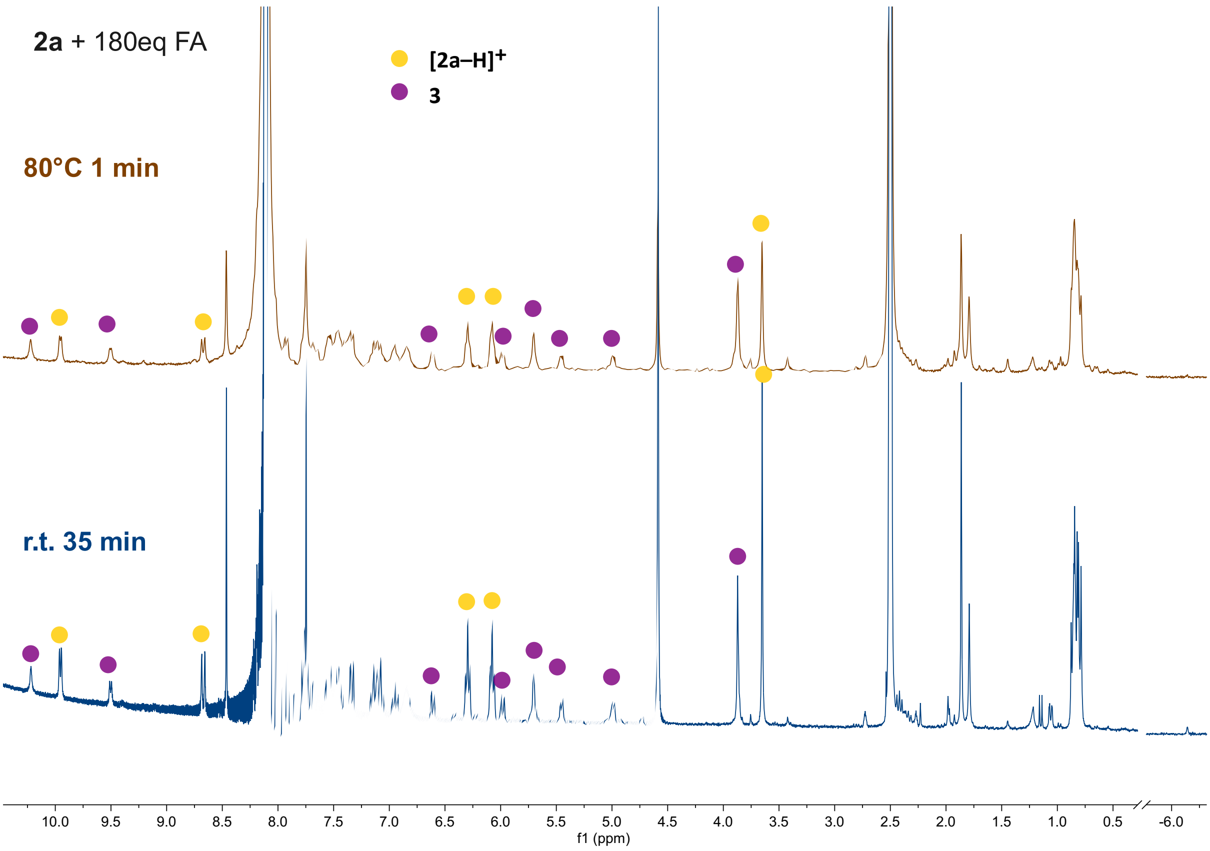

Figure S32. Stacked ^{1}H NMR spectra from Fig. S26 (bottom) and Fig. S31 (top) showing almost identical spectras at indicated timepoints. Signals assigned to **3** (•) and **[2a-H]**$^{+}$ (•) are marked.

7. Crystal Structure Determination

Crystals of **2a** and **[2a-H]**$^+$ were immersed in parabar oil, mounted at ambient conditions and transferred into the stream of nitrogen (173 K). A crystal of **2a** was measured on a *RIGAKU Synergy S* area-detector diffractometer[2] using mirror optics monochromated Cu *K*α radiation (λ = 1.54184 Å), while **[2a-H]**$^+$ was measured on an *Oxford Diffraction SuperNova* area-detector diffractometer[2] using mirror optics monochromated Mo *K*α radiation (λ = 0.71073 Å) and Al filtered.[6] The unit cell constants and an orientation matrix for data collection were obtained from a least-squares refinement of the setting angles of reflections in the range 2.382° < θ < 77.289° for **2a** and 2.023° < θ < 27.844° for **[2a-H]**$^+$. For **2a** a total of 4112 frames were collected using ω scans, with 3.2 second exposure time (10 s for high-angle reflections), a rotation angle of 0.5° per frame, a crystal-detector distance of 40.0 mm, at T = 173(2) K. For **[2a-H]**$^+$ a total of 834 frames were collected using ω scans, with 2.5+25 seconds exposure time, a rotation angle of 1.0° per frame, a crystal-detector distance of 65.0 mm, at T = 173(2) K.
Data reduction was performed using the *CrysAlisPro*[2] program. The intensities were corrected for Lorentz and polarization effects, and an absorption correction based on the multi-scan method using SCALE3 ABSPACK in *CrysAlisPro*[2] was applied. Data collection and refinement parameters are given in Table S2. The structures were solved by intrinsic phasing using *SHELXT*[3], which revealed the positions of all non-hydrogen atoms. All non-hydrogen atoms were refined anisotropically. H-atoms were assigned in geometrically calculated positions and refined using a riding model where each H-atom was assigned a fixed isotropic displacement parameter with a value equal to 1.2Ueq of its parent atom (1.5Ueq for methyl groups). Refinement of the structures was carried out on F^2 using full-matrix least-squares procedures, which minimized the function $\Sigma w(F_o^2 - F_c^2)^2$. The weighting schemes were based on counting statistics and included a factor to downweight the intense reflections. All calculations were performed using the *SHELXL-2014/7*[4] program in OLEX2[5].
The structure of **2a** was refined as a two-component twin, where the twin law corresponds to a rotation of -180 degrees around [-0.23 -0.00 0.97] (reciprocal) or [0.00 0.00 1.00] (direct). A disorder model was included for triflate, where the occupancy of the disorder components is refined using a free variable. The occupancies of both components together are restrained to 100%.
For **[2a-H]**$^+$, a disorder model was used for one of the PF_6 units and for the CH_2Cl_2. There is another disordered CH_2Cl_2 molecule, which could not be modeled and therefore a mask was used to include the contribution of the electron density located in the void area into the calculated structure factors.
Further crystallographic details are compiled in Table S2. Crystallographic data for both structures reported in this paper have been deposited with the Cambridge Crystallographic Data Centre (CCDC) as supplementary publication numbers 2251207 (**2a**), and 2251208 (**[2a-H]**$^+$).

Table S2. Crystal data and structure refinement for **2a** and **[2a-H]**$^+$.

Identification code	**2a**	**[2a-H]**$^+$
CCDC No	2251207	2251208
Empirical formula	$C_{32}H_{31}F_3N_4O_3RuS$	$C_{32}H_{34}Cl_2F_{12}N_4P_2Ru$
Formula weight	709.74	936.54
Temperature/K	173.01(10)	173.00(10)
Crystal system	monoclinic	monoclinic
Space group	$P2_1/c$	$P2_1/c$
a/Å	18.9076(3)	20.26618(17)
b/Å	11.49805(18)	10.02851(8)

c/Å	14.5081(2)	20.44489(17)
α/°	90	90
β/°	100.5586(15)	101.7636(9)
γ/°	90	90
Volume/Å^3	3100.67(9)	4067.94(6)
Z	4	4
ρ_{calc}g/cm^3	1.520	1.529
μ/mm^{-1}	5.220	0.679
F(000)	1448.0	1880.0
Crystal size/mm^3	0.22 × 0.154 × 0.049	0.316 × 0.216 × 0.095
Radiation	Cu Kα (λ = 1.54184)	Mo Kα (λ = 0.71073)
2Θ range for data collection/°	4.754 to 155.63	4.07 to 54.188
Index ranges	-23 ≤ h ≤ 23, -14 ≤ k ≤ 14, -16 ≤ l ≤ 18	-25 ≤ h ≤ 25, -12 ≤ k ≤ 12, -26 ≤ l ≤ 25
Reflections collected	12137	49828
Independent reflections	12137 [R_{int} = ?, R_{sigma} = 0.0132]	8903 [R_{int} = 0.0286, R_{sigma} = 0.0202]
Data/restraints/parameters	12137/6/458	8903/12/533
Goodness-of-fit on F^2	1.203	1.036
Final R indexes [I>=2σ (I)]	R_1 = 0.0766, wR_2 = 0.2636	R_1 = 0.0334, wR_2 = 0.0836
Final R indexes [all data]	R_1 = 0.0818, wR_2 = 0.2785	R_1 = 0.0397, wR_2 = 0.0874
Largest diff. peak/hole / e Å^{-3}	2.13/-0.97	0.83/-0.69

8. References

S1 N. Lentz, Y. Streit, P. Knörr and M. Albrecht, *Chem. Eur. J.*, 2022, **28**, e202202672.

S2 Oxford Diffraction, *CrysAlisPro, Version 1.171.40.37a. Oxford Diffraction Ltd., Yarnton, Oxfordshire, UK*, 2018.

S3 G. M. Sheldrick, *Acta. Crystallogr. A. Found. Adv.*, 2015, **71**, 3–8.

S4 G. M. Sheldrick, *Acta. Crystallogr. C. Struct. Chem.*, 2015, **71**, 3–8.

S5 O. V. Dolomanov, L. J. Bourhis, R. J. Gildea, J. A. K. Howard and H. Puschmann, *J. Appl. Crystallogr.*, 2009, **42**, 339–341.

S6 P. Macchi, H.-B. Bürgi, A. S. Chimpri, J. Hauser and Z. Gál, *J Appl Crystallogr*, 2011, **44**, 763–771.
